# NIK links inflammation to hepatic steatosis by suppressing PPARα in alcoholic liver disease

**DOI:** 10.7150/thno.40149

**Published:** 2020-02-18

**Authors:** Yaru Li, Mingming Chen, Yu Zhou, Chuanfeng Tang, Wen Zhang, Ying Zhong, Yadong Chen, Hong Zhou, Liang Sheng

**Affiliations:** 1 Department of Pharmacology, School of Basic Medical Science, Nanjing Medical University, Nanjing, Jiangsu 211166, China;; 2 Jiangsu Key Laboratory of Drug Screening, China Pharmaceutical University, Nanjing, Jiangsu 210009, China;; 3 Laboratory of Molecular Design and Drug Discovery, School of Science, China Pharmaceutical University, Nanjing, Jiangsu 211198, China;; 4 Department of Immunology, Nanjing Medical University, Nanjing, Jiangsu 211166, China;; 5 Key Laboratory of Rare Metabolic Diseases, Nanjing Medical University, Nanjing, Jiangsu 211166, China;; 6 Department of Rehabilitation Medicine, Jiangsu Province People's Hospital and Nanjing Medical University First Affiliated Hospital, Nanjing, Jiangsu 210029, China.

**Keywords:** carnitine palmitoyl transferase 1α, mitogen-activated protein kinase/extracellular signal-regulated kinase kinase, extracellular signal-regulated kinase, proinflammatory cytokine

## Abstract

**Background:** Inflammation and steatosis are the main pathological features of alcoholic liver disease (ALD), in which, inflammation is one of the critical drivers for the initiation and development of alcoholic steatosis. NIK, an inflammatory pathway component activated by inflammatory cytokines, was suspected to link inflammation to hepatic steatosis during ALD. However, the underlying pathogenesis is not well-elucidated.

**Methods:** Alcoholic steatosis was induced in mice by chronic-plus-binge ethanol feeding. Both the loss- and gain-of-function experiments by the hepatocyte-specific deletion, pharmacological inhibition and adenoviral transfection of NIK were utilized to elucidate the role of NIK in alcoholic steatosis. Rate of fatty acid oxidation was assessed *in vivo* and *in vitro*. PPARα agonists or antagonists of MEK1/2 and ERK1/2 were used to identify the NIK-induced regulation of PPARα, MEK1/2, and ERK1/2. The potential interactions between NIK, MEK1/2, ERK1/2 and PPARα and the phosphorylation of PPARα were clarified by immunoprecipitation, immunoblotting and far-western blotting analysis.

**Results:** Hepatocyte-specific deletion of NIK protected mice from alcoholic steatosis by sustaining hepatic fatty acid oxidation. Moreover, overexpression of NIK contributed to hepatic lipid accumulation with disrupted fatty acid oxidation. The pathological effect of NIK in ALD may be attributed to the suppression of PPARα, the main controller of fatty acid oxidation in the liver, because PPARα agonists reversed NIK-mediated hepatic steatosis and malfunction of fatty acid oxidation. Mechanistically, NIK recruited MEK1/2 and ERK1/2 to form a complex that catalyzed the inhibitory phosphorylation of PPARα. Importantly, pharmacological intervention against NIK significantly attenuated alcoholic steatosis in ethanol-fed mice.

**Conclusions:** NIK targeting PPARα via MEK1/2 and ERK1/2 disrupts hepatic fatty acid oxidation and exhibits high value in ALD therapy.

## Introduction

Ethanol intake is harmful at any dose and its risks rise with the increasing levels of consumption 1. Excessive drinking causes alcoholic liver disease (ALD), which covers a spectrum of pathological states encompassing alcoholic steatosis, alcoholic hepatitis, fibrosis, and cirrhosis 2. Alcoholic steatosis, defined histologically as the deposition of fat in small or large droplets in hepatocytes, is the initial phase of ALD 3. Excess fat accumulation in lipid droplets induces hepatocellular ballooning to hinder blood flow and microcirculation in sinusoidal space and consequently raises oxidative injury and endoplasmic reticulum stress in hepatocytes 4, 5. The latter two pathological events will aggravate ALD. Relieving alcoholic steatosis thus could be effective to prevent or delay the progression of fatal ALD.

Considerable evidence indicates that alcohol exposure weakens the intestinal barrier and facilitates the influx of lipopolysaccharide (LPS) 6, which increases the generation of reactive oxygen species 7. LPS and reactive oxygen species further stimulate Kupffer cells to secrete proinflammatory cytokines, such as tumor necrosis factor α (TNFα) 6 and interleukin 1β (IL1β) 8, which exacerbate inflammation and push the progression of alcoholic steatosis 9. Alcoholic steatosis is largely induced by the disruption of hepatic fatty acid oxidation 10; this disruption usually results from the damage to the function of peroxisome proliferator-activated receptor α (PPARα), a primary controller of fatty acid oxidation 11, 12. Fatty acid oxidation is critical to protect hepatic lipid homeostasis from excessive influx of fatty acids caused by alcohol-induced adipocyte lipolysis 13. However, there is little information concerning how inflammation affects hepatic fatty acid oxidation in the pathogenesis of alcoholic steatosis.

NF-κB-inducing kinase (NIK), a *Map3k14*-encoded serine/threonine kinase, is aberrantly activated in the livers of mice and patients with ALD 14, 15 due to cytokine and chemokine stimulation 16, 17. Locating at the upstream of noncanonical NF-κB pathway, NIK phosphorylates I-κB kinase α, subsequently initiating the phosphorylation and proteolytic cleavage of p100 (NF-κB2 precursor) to produce p52 (active NF-κB2 isoform) for the transcriptional regulation of target genes 18. NIK may be an opportunity to understand the role of inflammation in regulating alcoholic steatosis.

The present study revealed that NIK pushed aberrant fat accumulation in the liver by disrupting fatty acid oxidation during ALD, because NIK recruited and activated mitogen-activated protein kinase/extracellular signal-regulated kinase 1/2 (MEK1/2) and extracellular signal-regulated kinase 1/2 (ERK1/2) to suppress the fatty acid oxidation controller, PPARα. Therefore, NIK could be a therapeutic target to stop inflammation from promoting alcoholic steatosis.

## Methods

### Animals

All mouse experiments were conducted on the basis of relevant institutional and national guidelines. The experimental protocol (Protocol Number: 1704009-3) was approved by the Animal Care and Use Committee of Nanjing Medical University. NIK flox/flox mouse (*NIK^f/f^*) in C57BL/6 background, was a present from Professor Liangyou Rui (University of Michigan, Ann Arbor, MI, USA). The mouse was generated by inserting two loxp sites into intron 1 and intron 2 that flank exon 2-6 of *Map3k14*. To generate hepatocyte-specific NIK-deficient mice (*NIK^Δhep^*), we utilized *albumin*-cre transgenic mice (Jackson Laboratory, Bar Harbor, ME, USA) to cross with* NIK^f/f^* mice. Wild-type (WT) mice in C57BL/6 background were purchased from the Animal Core Facility of Nanjing Medical University, Nanjing, China. In a pathogen-free barrier facility with controlled temperature and illumination, mice had *ad libitum* access to sterile water and standard food. Following a previous study, male mice aged 10 weeks received chronic-plus-binge ethanol feeding 19. In detail as shown in Figure S1A, mice fed a Lieber-DeCarli ethanol diet (5% ethanol, Trophic Animal Feed High-Tech Co., Ltd, Haian, Jiangsu, China) for 10 d and thereafter were given a single gavage of ethanol (5 g/kg body weight) on the eleventh day. Fenofibrate was orally administered starting on the third day of ethanol feeding at a dose of 20 mg/kg/day 20. The NIK inhibitor B022, synthesized in accordance with a previous report 21, was dissolved in corn oil and intraperitoneally administrated starting on the third day of ethanol feeding at a dose of 25 mg/kg/day.

### Blood sample analysis

Blood was collected following decapitation. Serum levels of β-hydroxybutyrate were assayed using a kit (Megazyme International Ireland, Bray, Ireland).

### Cell culture and treatment

AML12 cells (SCSP-550) and HepG2 (SCSP-510) were purchased from the Cell Bank of the Chinese Academy of Sciences (Shanghai, China) and cultured according to the previous studies 22, 23. AML12 cells subjected to transfection were treated by stimuli 24 h latter, and then further cultured for another 24 h. Serum starvation started 5 h before harvest. HepG2 cells infected by adenovirus were further cultured for 24 h. Serum starvation started 5 h before harvest.

Primary hepatocytes were isolated by collagenase digestion from adult mice (8-10 weeks) according to a previously published protocol 15. After being cultured in William's medium E (Sigma-Aldrich, Shanghai, China) containing 6% fetal bovine serum (Lonsa, Richmond, VA, USA) for 16 h, hepatocytes were subjected to treatment with stimuli or adenoviral infection. Cells were harvested for subsequent assays after 24-hour culture.

### Plasmids

We purchased p3XFlag-CMV7.1 from Sigma-Aldrich and pcDNA3.1(+) from Invitrogen (Carlsbad, CA, USA). Dr. Dongping Wei (The First Hospital of Nanjing, Nanjing, Jiangsu, China) provided pcDNA-HA3, pcDNA-HA3-CPT1α (carnitine palmitoyl transferase 1α) and pcDNA-HA3-RXRα (retinoid-X receptor α). Dr. Liangyou Rui (University of Michigan Medical School) provided pRK5-NIK, pRK5-NIK(KA), and β-gal expression vectors. We subcloned pRK5-NIK and pRK5-NIK(KA) into pcDNA-HA3, pcDNA3.1(+), or pAdeno-TBG-MCS-3FLAG (OBiO Technology Corp., Ltd, Shanghai, China). Bruce Spiegelman provided pSG5 PPARα (Addgene plasmid #22751), which was subcloned into p3XFLAG-CMV7.1 (Sigma-Aldrich) and pcDNA-HA3. Vectors expressing PPARα mutants, including PPARα (S6, 12, 21A), PPARα (S73, 76, 77A) and PPARα (S6, 12, 21, 73, 76, 77A), were generated using p3XFLAG-CMV7.1-PPARα by Shanghai Generay Biotech Co., Ltd., Shanghai, China. A series of vectors expressing truncated PPARα (Figure S8) were prepared using pcDNA-HA3-PPARα. Bruce Spiegelman also provided pcDNA-f:PGC1 (peroxisome proliferator-activated receptor gamma coactivator 1, Addgene plasmid # 1026), which was subcloned into pcDNA-HA3. John Kyriakis provided pMT ERK1 (Addgene plasmid # 12656), which was subcloned into pcDNA-HA3. Melanie Cobb provided pCMV-myc-ERK2-MEK1_fusion (Addgene plasmid # 39194), from which an ERK2 fragment was subcloned into pcDNA-HA3, pCMV-3-tag-4A-myc, or pAdeno-MCMV-MCS-3FLAG (OBiO Technology Corp., Ltd.). PPRE X3-TK-Luc was provided by Bruce Spiegelman (Addgene plasmid # 1015). Fragments encoding mouse MEK1 and MEK2 were generated via polymerase chain reaction (PCR) from mouse liver cDNA and then inserted into pcDNA-HA3 or pCMV-3-tag-4A-myc. A HiSCript II 1st Strand cDNA Synthesis kit (Vazyme Biotech Co. Ltd, Nanjing, Jiangsu, China) was used to synthesize cDNA for cloning, and PCR was performed using Phanta Max Super-Fidelity DNA polymerase (Vazyme). Inserted fragments were cloned into vectors using a CloneExpress II One Step Cloning kit (Vazyme). The primers used for cloning are listed in Table S1.

### Generation of adenoviruses

Fragments encoding NIK or NIK(KA) were synthesized via PCR and inserted downstream of the *albumin* promoter in the pAdeno-TBG-MCS-3FLAG vector. The empty pAdeno-TBG-MCS-3FLAG vector and that containing the target genes were sent to OBiO Technology Corp., Ltd. for adenovirus packaging and purification.

### Adenoviral infection

Male mice aged 10 weeks were injected with adenoviruses (2 × 10^10^ viral particles [vp] for each mouse) through tail vein. At day 5 after infection, mice were decapitated after 18 h of fasting. Adenoviruses, with concentrations of 4 × 10^8^ vp/well in 12-well plates or 8 × 10^8^ vp/well in 6-well plates, infected primary hepatocytes and HepG2 cells. Cells were further cultured for 24 h before harvest.

### Immunoblotting and immunoprecipitation

We followed our previous protocol 24 to perform sample preparation, immunoblotting and immunoprecipitation. The FLAG-tagged proteins and HA-tagged proteins were immunoprecipitated by anti-FLAG M2 affinity gel (Sigma-Aldrich) and Pierce anti-HA agarose (Thermo Fisher Scientific, Shanghai, China), respectively. Cytosol and nuclear proteins were extracted by a kit (BioVision Incorporated, Milpitas, CA, USA). Bands in immunoblots were quantified using Image J software (National Institutes of Health, Bethesda, MD, USA; [1.37c]). The information concerning the antibodies and beads used is summarized in Table S2.

### Reverse transcriptional quantitative PCR

According to our previous protocol 24, we performed total RNA extraction, reverse-transcription and PCR. Ribosomal protein, large, P0 (RPLP0) and glyceraldehyde-3-phosphate dehydrogenase (GADPH) were internal controls for mouse samples and HepG2 cells, respectively. The primer pairs used for reverse transcriptional quantitative PCR in this study are listed in Table S3.

### Luciferase assay

AML12 cells were transfected with PPRE X3-TK-Luc (200 ng/well), β-gal expression vector (200ng/ well), and pSG5 PPARα (200 ng/well) plus other expression vectors (200 ng/well) as indicated using polyethylenimine (Sigma-Aldrich). Then, after growth for 24 h, cells received treatment of WY14643 (5μmol/ L, MedChemExpress), trametinib (100nmol/L, MedChemExpress), SCH772984 (50 nmol/L, MedChemExpress), or IKK16 (1 μmol/L, MedChemExpress) for another 24 h. Luciferase activity was determined with luciferase reporter assay system (Promega, Madison, WI, USA). β-gal activity, as the control for transfection efficiency, was measured by a kit (Mairybio Biological, Beijing, China). Results were averaged over three biological replicates.

### Histopathological analysis and liver triglyceride (TAG) assay

Staining by Hematoxylin and eosin (H&E) and Oil Red O (Sigma-Aldrich) were performed referring to the work of Sarmistha et. al. 25 to demonstrate lipid accumulation in the liver. Following our protocol 24, liver TAG was extracted by chloroform-methanol solution and determined by enzymatic method with kit.

### β-oxidation assays in hepatocytes

The β-oxidation rate was determined according to our previous study with minor modifications 26. Briefly, hepatocytes were incubated in serum-free William's medium E containing 100 μmol/L palmitate conjugated with bovine serum albumin and 2 μCi/mL [9, 10-^3^H] oleic acid (American Radiolabeled Chemicals, Inc., St. Louis, MO, USA) at 37 ℃ for 1 h. Then the culture media were transferred, mixed with perchloric acid (1.3 mol/L), and subjected to high-speed centrifugation. Thereafter, the supernatants were collected, neutralized by potassium hydroxide (2 mol/L) plus 3-(N-Morpholino) propanesulfonic acid (0.6 mol/L), and poured into an anion-exchange column (prepared with Dowex 1×8 anion-exchange resin, Aladdin, Shanghai, China) to get rid of ketone bodies. The radioactivity of ^3^H (tritium oxide) in the effluent normalized to protein amounts in the cells was determined to calculate β-oxidation rates.

### Far-western blotting

Far-western blotting was performed following a previously published protocol 27. The proteins that potentially interact with NIK, including PPARα, MEK1, and ERK2, were expressed with a HA tag in AML12 cells and purified by immunoprecipitation. These target proteins were transferred to polyvinylidene fluoride membrane by immunoblotting, subsequently probed by a purified GST-infused NIK protein (4 μg/mL, Promega, Madison, WI, USA), and eventually visualized using horseradish peroxidase (HRP)-conjugated GST tag monoclonal antibodies (#HRP-66001; Proteintech, Wuhan, China).

### Statistical analysis

Results are demonstrated as the means ± SEM. Two groups of data were compared by two-tailed Student's *t*-test. More than two groups of data were compared by one-way analysis of variance. p <0.05 indicates statistical significance.

## Results

### NIK mediates liver steatosis initiated by chronic-plus-binge ethanol feeding in mice

**Figure 1:**
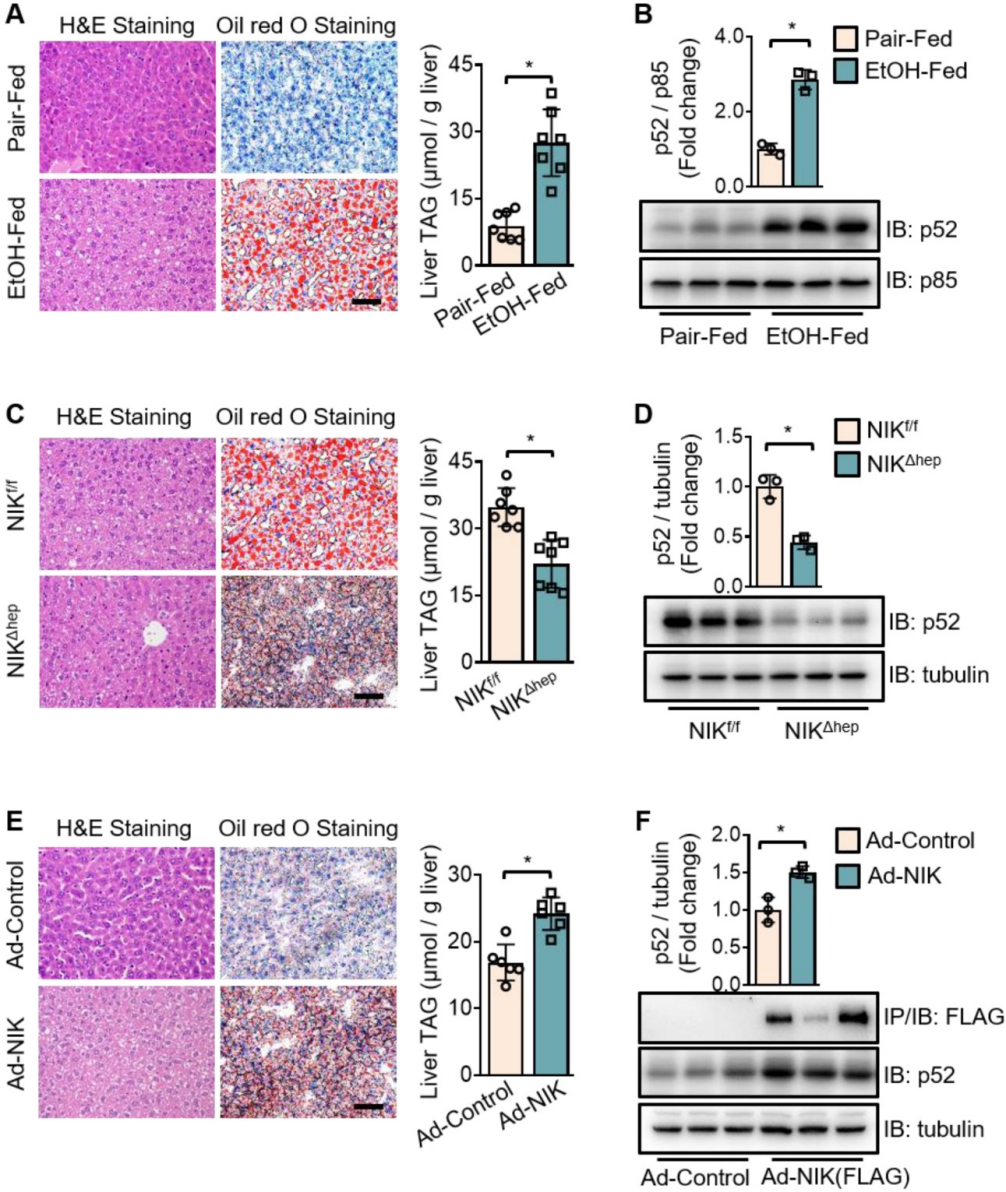
**Chronic-plus-binge ethanol feeding induces hepatic steatosis of mice by activating NIK in the liver.** (A, C, E) Representative staining of H&E and Oil Red O; liver TAG levels. Bar = 200 μm. (B, D, F) Representative immunoblots for FLAG-tagged NIK, p52, p85 and tubulin in the liver. (A, B) Wild-type (WT) mice received a chronic-plus-binge ethanol (EtOH-fed; n = 7) or control diet (Pair-fed; n = 7). (C, D) *NIK^f/f^* (n = 7) and *NIK^Δhep^* (n = 7) mice received chronic-plus-binge ethanol diets. (E, F) WT mice were infected with adenoviruses expressing FLAG-tagged NIK (Ad-NIK; n = 6) or control viruses (Ad-control; n = 6) for 5 d. Values are demonstrated as means ± SEM. *P <0.05, for comparisons with the control.

To investigate the role of NIK in alcoholic steatosis, we adopted an ALD mouse model of chronic-plus-binge ethanol feeding 19. As demonstrated in Figure S1A, mice received chronic ethanol feeding for 10 d and got an acute binge at day 11. In WT mice, ethanol feeding significantly increased the hepatic TAG content (Figure 1A) as well as p52 protein level (Figure 1B), a classic biomarker for NIK activation. Consistently, hepatic NIK protein level was also elevated by ethanol feeding (Figure S1C, upper panel) but the NIK mRNA level remained unchanged (Figure S1B). Furthermore, we found that both chronic ethanol feeding and acute binge contributed to the hepatic NIK upregulation (Figure S1C, lower panel). In hepatocyte-specific NIK-deficient mice (*NIK^Δhep^*), ethanol-induced TAG accumulation (Figure 1C) and aberrant p52 level increase (Figure 1D) in the livers were significantly reduced compared to those in WT control mice (*NIK^f/f^*). Overexpression of NIK significantly upregulated liver TAG and p52 levels (Figure 1E-F). These data indicate that NIK activity is associated with TAG levels in the liver and that NIK may be a driver of alcoholic steatosis.

To identify the NIK stimuli in intrahepatic environment of ALD, we utilized hepatocytes to test a series of factors under appropriate pathological concentrations. Judging by the protein levels of p52, we found that NIK was activated by hydrogen peroxide, palmitate, LPS, TNFα, and IL1β, but not by ethanol or its metabolites. Among these activators, LPS, TNFα, and IL1β exhibited the strongest activities (Figure S1D). LPS may activate NIK via Toll-like receptor pathway 28. TNFα and IL1β could enhance the stability of NIK protein 16, 17. That may be why ethanol feeding elevated NIK protein level but had no effect on NIK mRNA level in the liver.

### NIK promotes alcoholic steatosis via inhibition of fatty acid oxidation in the liver

**Figure 2:**
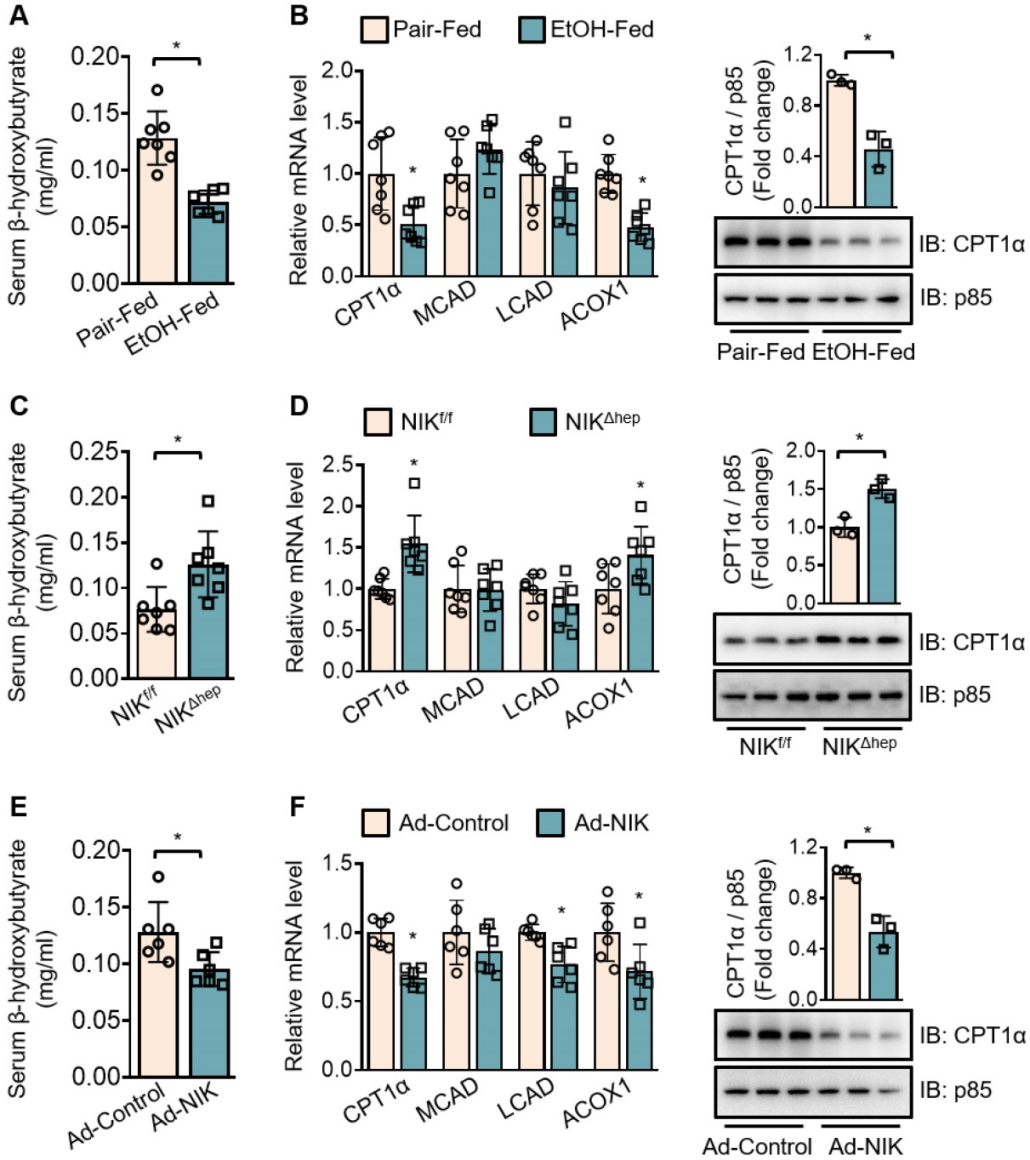
** Chronic-plus-binge ethanol feeding reduces serum levels of β-hydroxybutyrate and downregulates CPT1α by activating NIK in the liver.** (A, C, E) Serum levels of β-hydroxybutyrate. (B, D, F) The mRNA levels of CPT1α, MCAD, LCAD, ACOX1, and representative immunoblots of CPT1α in the liver. (A, B) WT mice received a chronic-plus-binge ethanol (EtOH-fed; n = 7) or control diet (Pair-fed; n = 7). (C, D) *NIK^f/f^* (n = 7) and *NIK^Δhep^* (n = 7) mice received chronic-plus-binge ethanol diets. (E, F) WT mice were infected with adenoviruses expressing FLAG-tagged NIK (Ad-NIK; n = 6) or control viruses (Ad-control; n = 6) for 5 d. Values are demonstrated as means ± SEM. *P <0.05, for comparisons with the control.

To evaluate the regulation of hepatic fatty acid oxidation by NIK during alcoholic steatosis, we determined the serum level of β-hydroxybutyrate and the expression of a series of oxidative genes (CPT1α; medium-chain acyl-coenzyme A dehydrogenase, MCAD; long-chain acyl-coenzyme A dehydrogenase, LCAD; acyl coenzyme A oxidase 1, ACOX1)− as indicators for hepatic fatty acid oxidation capacity. Ethanol consumption reduced the serum β-hydroxybutyrate level (Figure 2A) and downregulated the hepatic mRNA levels of CPT1α and ACOX1 (Figure 2B) in WT mice. *NIK^Δhep^* mice exhibited higher serum β-hydroxybutyrate levels (Figure 2C) as well as higher hepatic mRNA levels of CPT1α and ACOX1 (Figure 2D) than *NIK^f/f^* mice after ethanol feeding. Overexpression of NIK in the liver reduced serum β-hydroxybutyrate levels (Figure 2E) and CPT1α, LCAD, ACOX1 mRNA expression (Figure 2F). CPT1α, as a key enzyme in the mitochondrial oxidation of fatty acids, received our additional attention. The protein level of CPT1α changed in line with its mRNA level (Figure 2B, D and F), besides, the protein level of the exogenous recombinant CPT1α was not affected by co-expressed NIK in AML12 cells (Figure S1E). Hence, NIK should regulate CPT1α expression at the transcriptional instead of the post-transcriptional level. Taken together, these results indicate that NIK-mediated suppression of hepatic fatty acid oxidation should contribute to ethanol-induced TAG accumulation in the liver.

### NIK reduces fatty acid oxidation by suppressing hepatic PPARα

**Figure 3:**
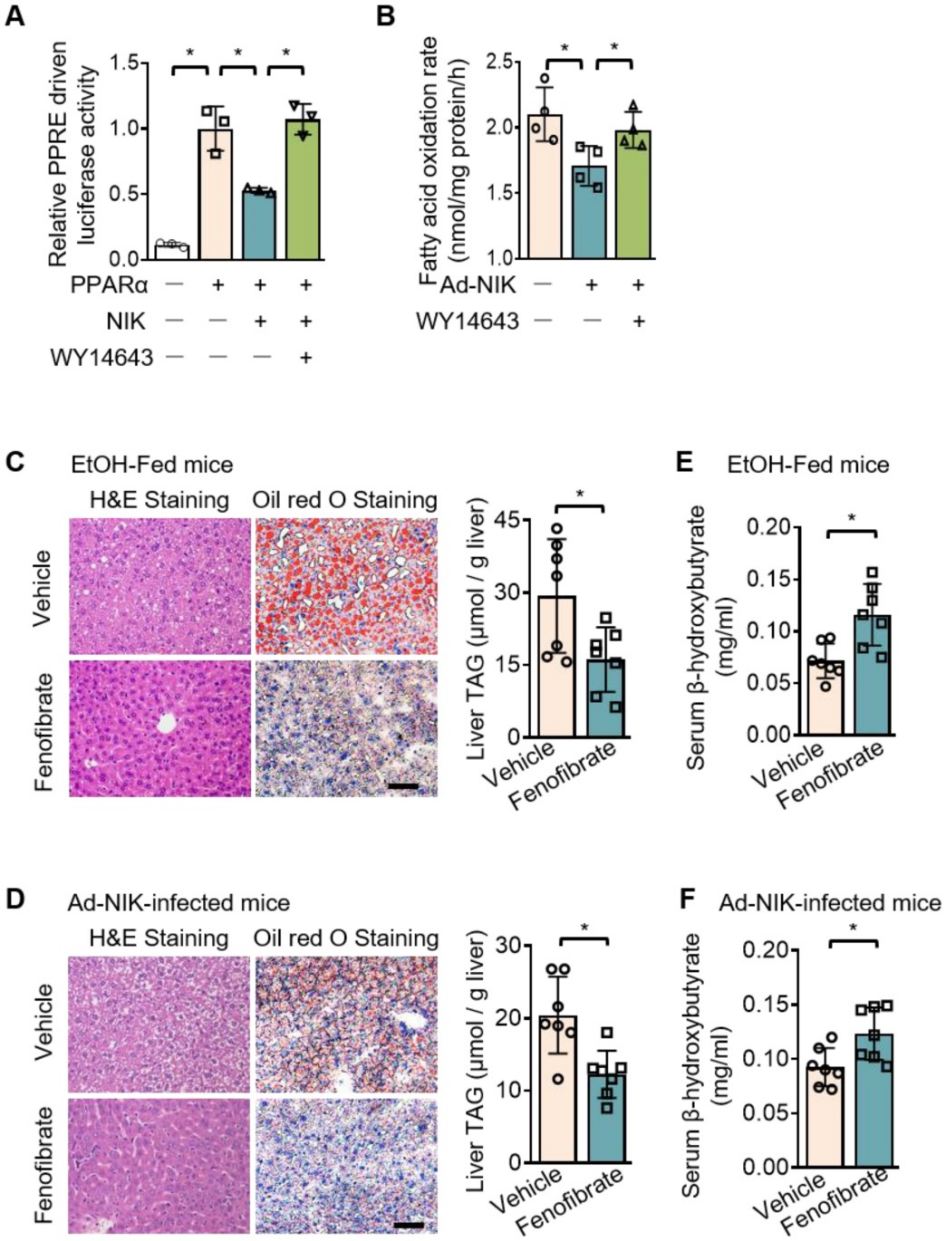
**NIK-induced hepatic steatosis and suppression of fatty acid oxidation are reversed by an agonist of PPARα.** (A) A luciferase assay assessing PPARα activity when NIK is overexpressed upon the treatment of WY14643 (5 μmol/L) (n=3 for each group). (B) Hepatocytes infected with adenoviruses expressing FLAG-tagged NIK (Ad-NIK; n = 4) or control adenoviruses (Ad-Control; n = 4) were exposed to WY14643 (5 μmol/L), and fatty acid oxidation rates were determined. (C, D) Representative staining of H&E and Oil Red O; liver TAG levels. Bar = 200 μm. (E, F) Serum levels of β-hydroxybutyrate. (C, E) WT mice fed with a chronic-plus-binge ethanol diet were treated with or without 20 mg/kg/day fenofibrates (n = 7 for treated and control groups). (D, F) WT mice infected with adenoviruses expressing FLAG-tagged NIK were treated with or without 20 mg/kg/day fenofibrates (n = 7 for treated and control groups). Values are demonstrated as means ± SEM. *P <0.05, for comparisons with the control.

A luciferase system was utilized to evaluate the role of NIK in the regulation of the transcriptional activity of PPARα, the primary controller of fatty acid oxidation in the liver 29. This luciferase system was successful in assessing PPARα activity, as the luciferase activity significantly increased when PPARα is overexpressed. NIK significantly reduced PPARα-driven luciferase activity, and WY14643, a high-performance PPARα selective agonist, protected PPARα activity from NIK (Figure 3A). NIK also suppressed fatty acid oxidation in hepatocytes, which was reversed by WY14643 (Figure 3B). In addition, the lowered mRNA and protein levels of CPT1α in hepatocytes due to NIK overexpression were reversed by WY14643 (Figure S2A). The NIK-induced suppression of fatty acid oxidation in hepatocytes was not caused by cell death because NIK overexpression did not reduce cell viability (Figure S2B). The regulation of PPARα by NIK was further assessed in mice treated with fenofibrate, a PPARα agonist used clinically. Hepatic steatosis induced by chronic-plus-binge ethanol feeding or NIK overexpression in the liver was significantly attenuated by fenofibrate (Figure 3C-D). Serum levels of β-hydroxybutyrate reduced by ethanol feeding or hepatic NIK overexpression were elevated by fenofibrate (Figure 3E-F), and correspondingly, the reduction in the mRNA and protein levels of CPT1α in the liver were reversed by fenofibrate (Figure S2C-D). These results suggest that PPARα is the regulatory node of NIK for pushing the process of alcoholic steatosis.

### NIK suppresses the transcriptional activity of PPARα by phosphorylation

**Figure 4:**
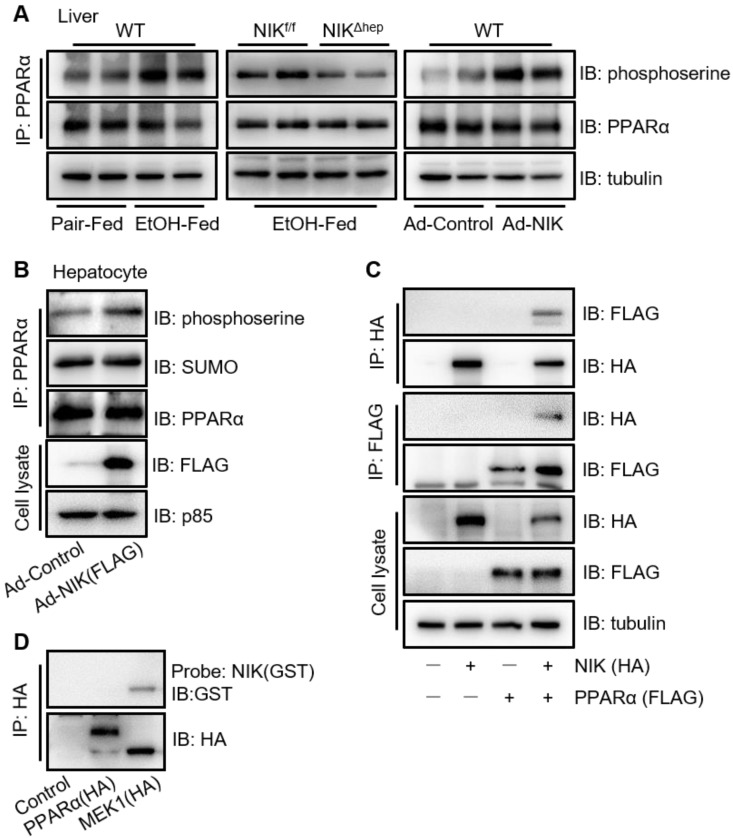
**NIK induces the phosphorylation of PPARα.** (A) WT mice were fed with a chronic-plus-binge ethanol or the control diet (left panel). *NIK^f/f^* and *NIK^Δhep^* mice were subjected to chronic-plus-binge ethanol feeding (middle panel). WT mice were infected with adenoviruses expressing NIK or control adenoviruses (right panel). Liver extracts were immunoprecipitated with an anti-PPARα antibody and immunoblotted with antibodies against phosphoserine, PPARα, or tubulin. (B) Hepatocytes isolated from WT mice were infected with adenoviruses expressing NIK (Ad-NIK) or control adenoviruses (Ad-Control). Cell extracts were immunoprecipitated with an anti-PPARα antibody and immunoblotted with antibodies against phosphoserine, SUMO, PPARα, or tubulin. (C) FLAG-tagged PPARα and HA-tagged NIK were coexpressed in AML12 cells. Cell extracts were immunoprecipitated with anti-FLAG M2 affinity gel or Pierce anti-HA agarose and immunoblotted with antibodies against HA, FLAG, or tubulin. (D) Enriched HA-tagged PPARα and MEK1 (positive control) were subjected to far-western blotting analysis using glutathione s-transferase (GST)-infused NIK as a probe and immunoblotted with antibodies against GST or HA.

To explore the regulatory mode of NIK on PPARα, we focused primarily on the phosphorylation of PPARα, as NIK is a kinase and it is reported that phosphorylation of the PPARα serine residues disrupts its transcriptional activity 30. Here, ethanol consumption increased the serine phosphorylation of PPARα in the liver of mice, which was attenuated by hepatocyte-specific NIK deletion (Figure 4A). Meanwhile, NIK overexpression enhanced the serine phosphorylation of PPARα in the liver and hepatocytes (Figure 4A-B). Small ubiquitin-like modifier (SUMO)-ylation, another inhibitory modification of PPARα 31, was not enhanced by NIK overexpression (Figure 4B). In addition, other regulatory modes were tested. Protein levels, and nuclear translocation of PPARα 32 in the liver were not affected by ethanol feeding, NIK deletion or NIK overexpression (Figure S3A). Surprisingly, the interactions between PPARα with its nuclear receptor heterodimer (RXRα) or its transcriptional coactivator (PGC1α) 32-34 were not reduced but enhanced by NIK overexpression (Figure S3B-C), implying that NIK-induced suppression was not via disrupting the interaction of PPARα with RXRα or PGC1α. NIK-activated canonical and noncanonical NF-κBs in hepatocytes by increasing the protein levels of p52 and (v-rel reticuloendotheliosis viral oncogene homolog A) RelA in nuclei (Figure S3D) theoretically may suppress PPARα transcriptional activity by interfering with the binding of PPARα to DNA 35. However, IKK16, the inhibitor of I-κB kinase α and I-κB kinase β that completely blocked NF-κB activation, did not significantly reverse the inhibition of PPARα activity or fatty acid oxidation by NIK (Figure S3E-F). This suggests that, in hepatocytes, NIK-NF-κB axis provides negligible contribution to the suppression of PPARα, and phosphorylation is the primary mode employed by NIK to regulate PPARα. To identify the mechanism underlying the ability of NIK to phosphorylate PPARα, we confirmed the interaction of NIK with PPARα by immunoprecipitation (Figure 4C). However, NIK did not directly bind to PPARα, as indicated by far-western blotting analysis (Figure 4D). It implies that NIK and PPARα coexist in one complex, but NIK-induced phosphorylation of PPARα relies on other kinases.

### NIK induces the phosphorylation of PPARα via MEK1/2 and ERK1/2

**Figure 5:**
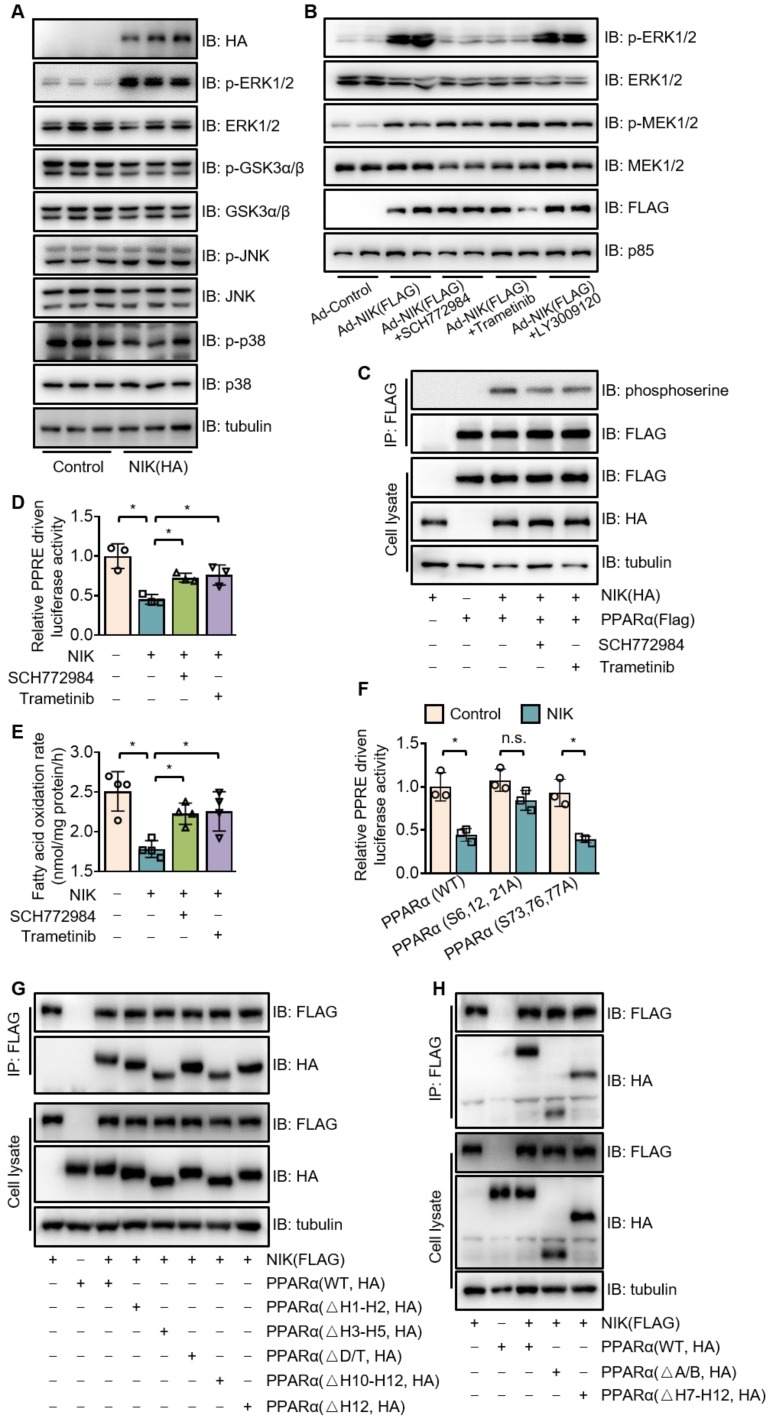
** NIK induces the phosphorylation of PPARα via the MEK1/2-ERK1/2 pathway.** (A) The extracts of AML12 cells, transfected with a vector expressing HA-tagged NIK or a control vector, were blotted with antibodies as indicated. (B) Hepatocytes were infected with NIK (Ad-NIK) or control (Ad-Control) adenoviruses and simultaneously treated with 50 nmol/L SCH772984, 100 nmol/L trametinib, 500 nmol/L LY3009120, or a vehicle. Cell extracts were blotted using antibodies as indicated. (C) AML12 cells, expressing HA-tagged NIK and FLAG-tagged PPARα, were treated with 50 nmol/L SCH772984 or 100 nmol/L trametinib. Cell extracts were immunoprecipitated with an anti-FLAG M2 affinity gel and immunoblotted with antibodies against phosphoserine, FLAG, HA, or tubulin. (D) Results of luciferase assays assessing PPARα activity when NIK and PPARα is overexpression with or without the treatment with SCH772984 (50 nmol/L) or trametinib (100 nmol/L; n = 3 for each group). (E) Hepatocytes infected with adenoviruses expressing FLAG-tagged NIK (Ad-NIK) with or without the treatment with SCH772984 (50 nmol/L) or trametinib (100 nmol/L; n = 4 for each group). The fatty acid oxidation rates were determined. (F) Results of luciferase assays assessing PPARα activity under coexpression of NIK with PPARα(WT) or PPARα(S6,12,21A) or PPARα(S73,76,77A) (n = 3 for each group). (G, H) Flag-tagged NIK was coexpressed with HA-tagged PPARα truncations as indicated in AML12 cells. Cell extracts were immunoprecipitated with an anti-FLAG M2 affinity gel and immunoblotted with antibodies against FLAG, HA, or tubulin. Values are demonstrated as means ± SEM. *P <0.05, for comparisons with the control.

To identify the mediators implicated in PPARα phosphorylation by NIK, a series of currently known kinases that catalyze inhibitory phosphorylation of PPARα were screened 36. Among them, ERK1/2 was verified to be the unique kinase activated by NIK via phosphorylation (Figure 5A). To authenticate the way by which NIK phosphorylates ERK1/2, we utilized inhibitors against Raf-1 (LY3009120), MEK1/2 (trametinib), and ERK1/2 (SCH772984) to disrupt the Raf-1-MEK1/2-ERK1/2 pathway. As shown in Figure 5B, trametinib and SCH772984, but not LY3009120, prevented ERK1/2 phosphorylation caused by NIK; besides, LY3009120 did not reduce NIK-induced MEK1/2 phosphorylation. These data indicated that MEK1/2 was a necessary pathway for NIK to induce ERK1/2 phosphorylation, and NIK phosphorylated MEK1/2 without involvement of Raf-1. Consistently, in the liver, the phosphorylation levels of MEK1/2 and ERK1/2 were elevated during NIK activation in the case of ethanol feeding and NIK overexpression, but were attenuated when NIK was deleted (Figure S4). To elucidate the way of NIK to interact MEK1/2 and ERK1/2, coimmunoprecipitation and far-western blotting were used. As shown by coimmunoprecipitation (Figure S5A-B), NIK coexisted with MEK1/2 and ERK1/2 in one complex. Based on the structural and functional similarities between MEK1 and MEK2 as well as between ERK1 and ERK2, MEK1 and ERK2 were selected for far-western blotting assay as the representatives of MEK1/2 and ERK1/2, respectively. The data indicated that NIK directly bound MEK1 and ERK2 (Figure S5C). It suggests that NIK recruits MEK1/2 and ERK1/2 in a direct combination to form a ternary complex.

Besides the interaction with NIK, MEK1/2 and ERK1/2 also bound PPARα (Figure S6). To confirm the mediating effect of ERK1/2 and MEK1/2 on NIK-induced phosphorylation and suppression of PPARα, the inhibitors of MEK1/2 (trametinib) and ERK1/2 (SCH772984) were used. Trametinib and SCH772984 attenuated PPARα phosphorylation (Figure 5C) and revised the PPARα activity (Figure 5D) and fatty acid oxidation (Figure 5E) suppressed by NIK. The ERK1/2-targeted phosphorylation sites in PPARα are serine residues, including S6, S12, S21, S73, S76 and S77 30, and PPARα mutants, such as PPARα (S6, 12, 21A), PPARα (S73, 76, 77A) and PPARα (S6, 12, 21, 73, 76, 77A) were prepared by mutating relevant serine residues into alanine residues. That NIK induced the phosphorylation of those serine residues were verified, as the PPARα mutants, PPARα (S6, 12, 21A) and PPARα (S6, 12, 21, 73, 76, 77A), had significantly lower phosphorylation levels compared to the wildtype one under NIK coexpression (Figure S7). PPARα (S6, 12, 21A) but not PPARα (S73, S76, S77A) was resistant to NIK-mediated suppression (Figure 5F), suggesting that S6, S12 and S21 are responsible for NIK's regulation over PPARα. Besides those ERK1/2-targeted serine residues, NIK actually induced phosphorylation on other sites of PPARα, since PPARα with all the ERK1/2-targeted sites mutated could still be phosphorylated by NIK (Figure S7).

PPARα is consist of four functional domains, including A/B, C, D, and E/F 37, and the C-terminal of D region plus E/F region contain 12 helices (H1-H12) 38. The motifs, so-called D-box and T-box located in C region and D region, respectively, are involved in heterodimerization 39. H1-H2 in D region plays a role in the interaction with corepressors 40. The E/F region also has motifs involved in heterodimerization and interaction with coactivator /corepressor, including H3-H5, H7-H9, H10-H11 (overlapping leucine-zipper region), H12 (overlapping LLXXLL-binding pocket) 38, 39. To identify the functional motifs of PPARα to interact the NIK-MEK1/2-ERK1/2 ternary complex, we constructed a series of vectors expressing truncated PPARα lacking A/B domain, D/T-box, H1-H2 region, H3-H5 region, H7-H12 region, H10-H12 region, or H12 region (Figure S8). The Coimmunoprecipitation indicated that PPARαΔA/B, PPARαΔH3-H5, PPARαΔH7-H12, PPARαΔH10-H12 showed lower affinity for NIK compared to PPARαWT, while, the affinity of PPARαΔD/T, PPARαΔH1-H2, and PPARαΔH12 for NIK did not decrease (Figure 5G-H). Besides, PPARαΔH7-H12 and PPARαΔH10-H12 showed a similar degree of decline in affinity for NIK (reduced by around 30%). These results suggest that A/B domain, H3-H5 region, and H10-H11 region are involved in the interaction of PPARα and the NIK-recruited complex.

To determine whether NIK regulates PPARα in an analogous manner in human and mouse hepatocytes, we utilized a hepatocyte cell line from human, HepG2. Similarly in mouse primary hepatocytes and liver, NIK overexpression reduced the rate of fatty acid oxidation and the mRNA levels of related genes, and enhanced the phosphorylation levels of MEK1/2, ERK1/2 and PPARα (Figure S9). Slightly differently, NIK overexpression reduced the protein levels of MEK1/2 and ERK1/2, which were not observed in mouse hepatocyte or liver (Figure S4 and S9C). These data suggest that NIK also inhibits fatty acid oxidation and PPARα through NIK-MEK1/2-ERK1/2 pathway in human liver cells.

### NIK phosphorylates PPARα in kinase activity-dependent and -independent manners

**Figure 6:**
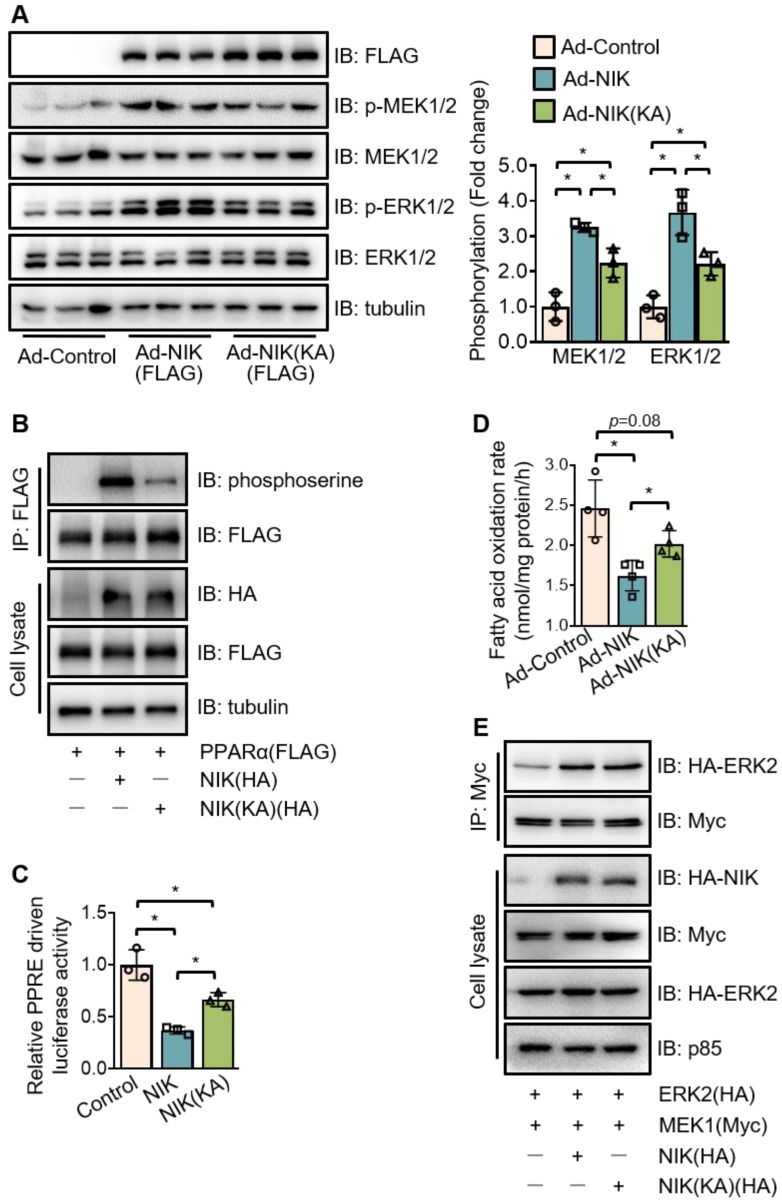
**NIK induces the phosphorylation of MEK1/2, ERK1/2, and PPARα in kinase activity -dependent and -independent manners.** (A) Hepatocytes isolated from WT mice were infected with adenoviruses expressing FLAG-tagged NIK (Ad-NIK) or NIK(KA) (Ad-NIK[KA]) or control adenoviruses (Ad-Control) for 24 h. Cell extracts were immunoblotted with antibodies as indicated. (B) The extracts of AML12 cells expressing FLAG-tagged PPARα with HA-tagged NIK or NIK(KA) were immunoprecipitated with an anti-FLAG M2 affinity gel and immunoblotted with antibodies against phosphoserine, FLAG, HA, and tubulin. (C) Results of luciferase assays assessing PPARα activity when NIK or NIK(KA) is overexpressed with PPARα (n = 3 for each group). (D) Hepatocytes infected with adenoviruses expressing FLAG-tagged NIK (Ad-NIK) or NIK(KA) (Ad-NIK[KA]) or control adenoviruses (Ad-Control) (n = 4 for each group). The fatty acid oxidation rates were determined. (E) AML12 cells expressing Myc-tagged MEK1 and HA-tagged ERK2 with HA-tagged NIK or NIK(KA). Cell extracts were immunoprecipitated with an anti-Myc antibody and immunoblotted with antibodies against Myc, HA or tubulin. Values are demonstrated as means ± SEM. *P <0.05, for comparisons with the control.

To further evaluate the role of NIK in PPARα phosphorylation, the kinase deficient mutant of NIK, NIK(KA), was compared with the WT one. Interestingly, NIK(KA), under the similar expression level with NIK, did not lose but retain the ability to induce the phosphorylation of MEK1/2, ERK1/2, and PPARα at a reduced level (Figure 6A-B). Therefore, NIK(KA) kept some of the inhibitory actions of NIK, and exhibited a weaker suppression of PPARα activity and fatty acid oxidation (Figure 6C-D). These results imply that NIK phosphorylates MEK1/2, ERK1/2, and PPARα and inhibits hepatocyte fatty acid oxidation in kinase activity-dependent and -independent manners. As NIK directly bound MEK1 and ERK2 rather than PPARα in the complex (Figure 4C-D, Figure S5 and Figure S6), the regulatory modes dependent or independent of NIK kinase activity may be largely due to NIK's regulation of MEK1/2 and ERK1/2. To test this hypothesis, the interaction between MEK1 and ERK2 was assessed under overexpression of NIK or NIK(KA). NIK enhanced the interaction of MEK1 with ERK2, which was sustained by NIK(KA) (Figure 6E). It suggests that besides directly phosphorylating MEK1/2 or ERK1/2, NIK may recruit MEK1/2 and ERK1/2 to enhance their interaction and thus facilitate the phosphorylation of ERK1/2 by MEK1/2.

### Pharmacological intervention against NIK attenuates alcoholic steatosis

**Figure 7:**
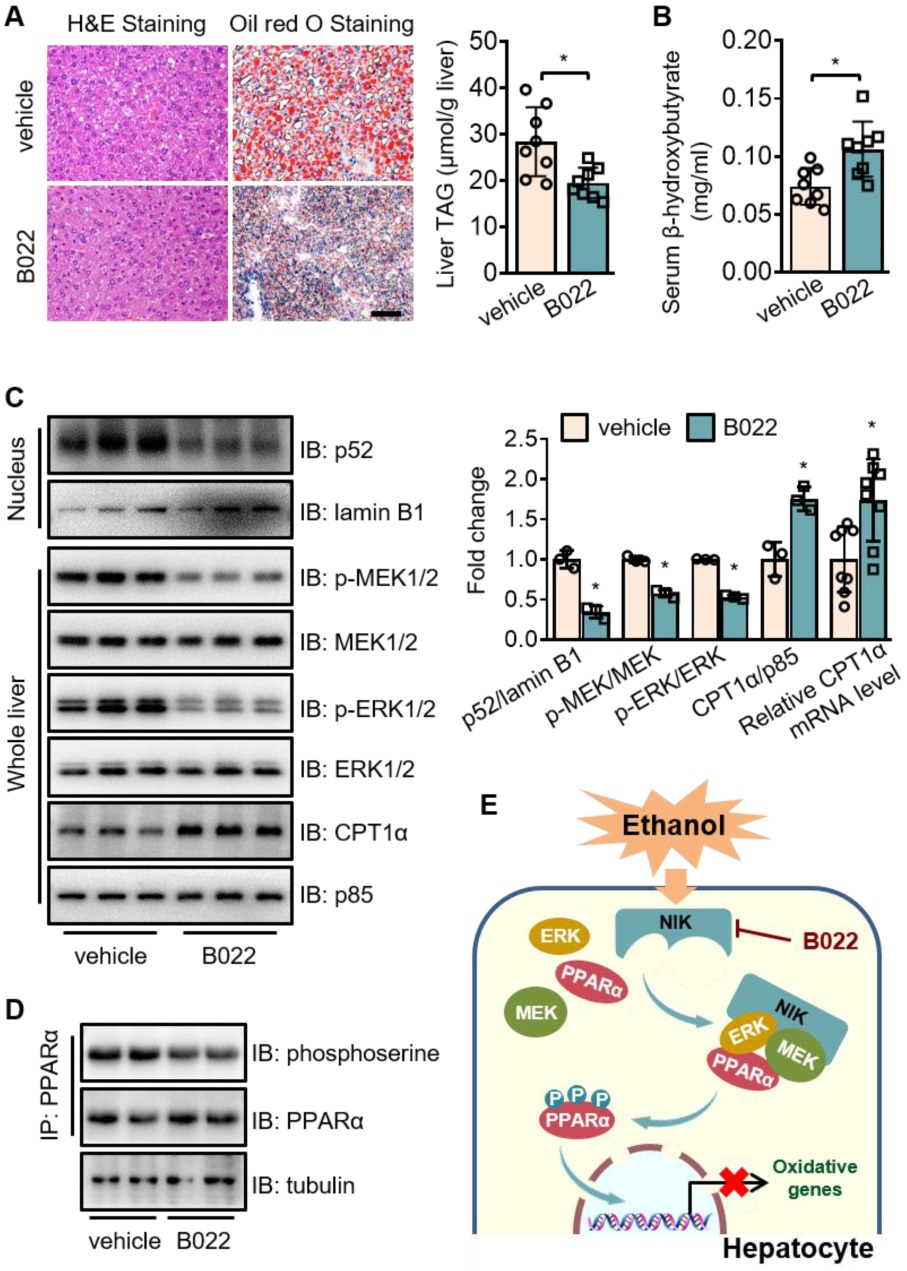
** B022, a NIK inhibitor, protects against ethanol-induced hepatic steatosis in mice.** WT mice fed with a chronic-plus-binge ethanol diet were administered with B022 (25 mg/kg/day) intraperitoneally starting on the third day of ethanol feeding (n = 8 for each group). (A) Representative staining of H&E and Oil Red O; liver TAG levels. Bar = 200 μm. (B) Serum level of β-hydroxybutyrate. (C) Representative immunoblots of p52, lamin B1, p-MEK1/2, MEK1/2, p-ERK1/2, ERK1/2, CPT1α, and p85 in the liver; the mRNA levels of hepatic CPT1α. (D) Liver extracts were immunoprecipitated with an anti-PPARα antibody and immunoblotted with antibodies against phosphoserine, PPARα, or tubulin. Values are demonstrated as means ± SEM. *P <0.05, for comparisons with the control. (E) Proposed model for NIK action during the pathogenesis of alcoholic steatosis. Ethanol consumption activates hepatic NIK, whose activity is suppressed by B022. NIK induces the inhibitory phosphorylation of PPARα by recruiting MEK1/2, ERK1/2, and PPARα, which prevent the PPARα-mediated transcription of genes related to fatty acid oxidation.

To assess the therapeutic value of NIK for ALD treatment, we used B022, a chemical NIK inhibitor, to treat mice fed with chronic-plus-binge ethanol diet. B022 attenuated ethanol-induced hepatic steatosis (Figure 7A), elevated β-hydroxybutyrate level in serum (Figure 7B), and enhanced CPT1α mRNA and protein levels in the liver (Figure 7C). B022 also relieved NIK activation, blocked MEK1/2-ERK1/2 pathway and protected PPARα activity in the liver as shown by the reduced nuclear levels of p52 as well as the decreased phosphorylation levels of MEK1/2, ERK1/2, and PPARα (Figure 7C-D). Therefore, B022 successfully protected the hepatic capacity of fatty acid oxidation from ethanol feeding and attenuated alcoholic steatosis by repressing the NIK-MEK1/2-ERK1/2 pathway.

## Discussion

In the present study, we have presented evidence that NIK is responsible for liver steatosis induced by chronic-plus-binge ethanol feeding in mice. Deletion of NIK in hepatocytes attenuated liver steatosis after ethanol consumption by protecting the hepatic capacity of fatty acid oxidation. PPARα, the primary transcriptional controller of fatty acid oxidation, is a regulatory node of NIK, as PPARα agonists reverse NIK-mediated hepatic steatosis and malfunction of fatty acid oxidation. NIK recruits MEK1/2 and ERK1/2 to form a complex that induces the inhibitory phosphorylation of PPARα (Figure 7F). Pharmacological intervention of NIK resulted in a prominent therapeutic effect on alcoholic steatosis. Therefore, NIK may be a valuable therapeutic target for treating ALD.

The influx of gut-derived LPS induced by ethanol consumption initiates inflammation during ALD. LPS stimulates Kupffer cells to secrete proinflammatory cytokines and chemokines, such as TNFα and IL1β 6, 8, 41. TNFα, IL1β, and LPS exhibit much higher efficiency to activate hepatocyte NIK than ethanol and its metabolites. Moreover, preventing gut-derived LPS flux by depleting intestinal microflora with antibiotics significantly attenuated alcoholic steatosis 6. These prove that enterohepatic axis-derived inflammation, as the most essential cause of NIK activation, plays a key role in the development of ALD. Because hepatocyte NIK deletion attenuated alcoholic steatosis, NIK likely links inflammation to ethanol-induced liver steatosis. The efficacy of the treatment of alcoholic steatosis by a NIK inhibitor, B022, further confirms that NIK has potential as a therapeutic target for ALD. However, NIK deficiency in hepatocytes failed to improve nonalcoholic steatosis induced by a high-fat diet 42, despite that NIK is activated both in nonalcoholic and alcoholic steatosis 14, 15. These results are presumably attributed to the differences in pathogenesis between the two fatty liver models. Excessive adipocyte lipolysis induced by ethanol exposure 13 leads to alcoholic steatosis when fatty acid oxidation is disrupted by aberrantly activated NIK in hepatocytes, whereas high-fat diet feeding induces chronic TAG deposits largely through the enhancement of hepatic lipogenesis resulting from the synergy and complementarity of the NIK pathways in multiple liver cell types besides hepatocytes 42. Therefore, simply deleting NIK in hepatocytes is enough to attenuate alcoholic steatosis by rescuing fatty acid oxidation but is insufficient to reverse high-fat diet-induced excessive lipogenesis contributed by diverse liver cell types. It is likely that NIK is the better therapeutic target for alcoholic steatosis than for nonalcoholic steatosis.

PPARα, the main controller of fatty acid oxidation in the liver, is regulated by NIK during ALD, considering that PPARα agonists reversed NIK-mediated suppression of PPARα activity, reduction in fatty acid oxidation, and hepatic steatosis. After screening several potential regulatory modes, we determine that serine phosphorylation contributes to the suppression of PPARα by NIK, as PPARα with triple mutations at S6, S12 and S21 is resistant to NIK-mediated suppression. However, there are conflicted reports for the activity regulation of PPARα by the phosphorylation of those serine residues. Juge-Aubry, C. E., et al. believed that the phosphorylation at S12 and S21 enhanced PPARα activity 43, but Barger PM, et al. demonstrated that phosphorylation at S6, S12 and S21 suppressed PPARα activity 30. Our results were consistent to the latter. As PPARα activity was regulated by a series of coactivators or corepressors 40, 44-46, we speculate that phosphorylation of S6, S12, S21 may change the configuration of PPARα leading to the dissociation of some corepressor, which may facilitate the entry of other coactivator or corepressor. Whether coactivator or corepressor binds to PPARα may depend on the cell state. That should be why phosphorylation at those residues causes different regulation of PPARα activity 30, 43. In case NIK is activated, NIK may recruit some potent corepressor facilitating the interaction of corepressor with PPARα and thus suppress PPARα activity. Of course, these hypotheses require further confirmation.

NIK does not directly phosphorylate PPARα. It integrates MEK1/2, ERK1/2, and PPARα into a complex, in which, NIK directly binds MEK1/2 and ERK1/2 but not PPARα, therefore, the phosphorylation of PPARα by NIK should be conducted through MEK1/2 and ERK1/2. ERK1/2 is the unique kinase phosphorylated and activated by NIK among those reported to catalyze inhibitory phosphorylation of PPARα 30, 36. MEK1/2, the upstream kinase of ERK1/2 and substrate of NIK 47, has been confirmed necessary for NIK-induced ERK1/2 phosphorylation. Thus, the phosphorylation should be transmitted along the NIK-MEK1/2-ERK1/2 pathway to PPARα. The A/B domain, H3-H5 region, and H10-H11 region in PPARα were verified contributable to the interaction of PPARα and NIK-MEK1/2-ERK1/2 complex. The A/B region contributing 70% transcriptional activity of PPARα contains an activation function-1 domain, which forms an amphiphilic α-helix probably interacting with coactivator or corepressor 48, and NIK-induced phosphorylation also occurs in A/B region. H3-H5 region plays a role in the heterodimerization with RXRα 49, suggesting that part of interaction between PPARα and the NIK-recruited complex could involve RXRα. H10-H11 region contains leucine-zippers, which is a putative docking motif of ERK1/2 39. These data imply that NIK-recruited complex anchors PPARα at multiple sites, and these sites are not necessarily close to phosphorylation sites of PPARα.

Of note, the phosphorylation of MEK1/2 and ERK1/2 is not entirely driven by NIK kinase activity, as this phosphorylation catalytic capacity of NIK is partially retained by its kinase deficient mutant, NIK(KA). We speculate that the integrating function of NIK may be also contributable, because NIK and NIK(KA) have a similar ability to enhance the interaction between MEK1 and ERK2, which may promote spatial proximity of MEK1 to ERK2 and thus facilitate the phosphorylation of ERK2 by MEK1. In fact, MEK1/2 is also the substrate of ERK1/2, however, ERK1/2 catalyzes an inhibitory phosphorylation of MEK1/2 on threonine residues at positions 292 or 386 50 instead of the activating phosphorylation on serine residues at positions 217 and 221 that were detected by the phospho-MEK1/2 antibody used in the present study. Hence, the increase in activating phosphorylation of MEK1/2 could be conducted by some NIK(KA)-recruited kinase but not ERK1/2. To identify this unknown kinase recruited by NIK is conducive to further understand NIK function, and the corresponding regulatory mechanism remains to be explored.

NIK is constitutively subjected to degradation mediated by a complex that includes cellular inhibitor of apoptosis 1/2 and tumor necrosis factor receptor-associated factors 2/3 in a ubiquitination/ proteasome-dependent manner 16, 17, 51. Cytokine stimulation blocks this degradation process, leading to NIK stabilization and activation 16, 17. As NIK phosphorylates and suppresses PPARα in kinase activity-dependent and -independent manners, accelerating NIK degradation is expected to be more effective than simply inhibiting NIK activity in the treatment of ALD or other NIK-MEK1/2-ERK1/2 complex-involved diseases. In addition to regulating lipid-metabolism genes, NIK seems to control a complex program coordinating the expression and secretion of proinflammatory factors, which sustain or amplify the inflammatory states of the liver. Specifically, NIK stimulates hepatocytes to release proinflammatory factors that trigger immune cell activation via a paracrine mechanism. Immune cell-generated proinflammatory factors further activate more immune cells to exacerbate liver inflammation 10. The liver inflammation may in turn stimulate NIK in hepatocytes to deteriorate the disorder in lipid metabolism during ALD. Thus, NIK suppression has dual effects of anti-inflammation and anti-fat deposition in the liver, and these effects are both beneficial to revise alcoholic steatosis in ALD therapy.

In conclusion, the aberrant activation of NIK may represent the underlying pathogenesis of alcoholic steatosis in mice. NIK plays a causal role in impeding fatty acid oxidation by restraining PPARα activity in hepatocytes. We identified the NIK-MEK1/2-ERK1/2 pathway as the main signaling route through which NIK regulates PPARα. Our study suggests that disruption of NIK in hepatocytes could offer new therapeutics for the treatment of ALD.

## Supplementary Material

Supplementary figures and tables.

## Abbreviations

ACOX1: acyl coenzyme A oxidase 1
ALD: alcoholic liver disease
Ad: adenovirus
CPT1α: carnitine palmitoyl transferase 1α
ERK: extracellular signal-regulated kinase
EtOH: ethanol
GADPH: glyceraldehyde-3-phosphate dehydrogenase
HRP: horseradish peroxidase
IB: immunoblot
IL1β: interleukin1 β
IP: immunoprecipitation
LCAD: long-chain acyl-coenzyme A dehydrogenase
LPS: lipopolysaccharide
MCAD: medium-chain acyl-coenzyme A dehydrogenase
MEK: mitogen-activated protein kinase/extracellular signal-regulated kinase kinase
mut: mutated
NIK: NF-κB-inducing kinase
n.s.: no significance
PGC1α: peroxisome proliferator-activated receptor gamma coactivator 1α
PPARα: peroxisome proliferator-activated receptor α
PPREs: PPAR response elements
RelA: v-rel reticuloendotheliosis viral oncogene homolog A
PCR: polymerase chain reaction
RXRα: retinoid-X receptor α
RPLP0: ribosomal protein, large, P0
SDS-PAGE: sodium dodecyl sulphate-polyacrylamide gel electrophoresis
SEM: standard error of the mean
SUMO: small ubiquitin-like modifier
TAG: triglyceride
TNFα: tumor necrosis factor α
vp: viral particles
WT: wild-type
