# Supplementary material for: NIK links inflammation to hepatic steatosis by suppressing PPARα in alcoholic liver disease
Source: Theranostics. 2020 Feb 18;10(8):3579–93. doi: 10.7150/thno.40149 (PMC7069072; doi:10.7150/thno.40149)
Supplement: Supplementary file 1 — Supplementary figures and tables. [file thnov10p3579s1.pdf]

Supplementary Information

NIK links inflammation to hepatic steatosis by suppressing PPARα in alcoholic liver disease

Yaru Li[1]*, Mingming Chen[2]*, Yu Zhou[1], Chuanfeng Tang[1], Wen Zhang[1], Ying Zhong[1], Yadong Chen[3], Hong Zhou[4], Liang Sheng[1,5,6 §]

[1]Department of Pharmacology, School of Basic Medical Science, Nanjing Medical University, Nanjing, Jiangsu 211166, China;

[2]Jiangsu Key Laboratory of Drug Screening, China Pharmaceutical University, Nanjing, Jiangsu 210009, China;

[3]Laboratory of Molecular Design and Drug Discovery, School of Science, China Pharmaceutical University, Nanjing, Jiangsu 211198, China;

[4]Department of Immunology, Nanjing Medical University, Nanjing, Jiangsu 211166, China;

[5]Key Laboratory of Rare Metabolic Diseases, Nanjing Medical University, Nanjing, Jiangsu 211166, China;

[6]Department of Rehabilitation Medicine, Jiangsu Province People's Hospital and Nanjing Medical University First Affiliated Hospital, Nanjing, Jiangsu 210029, China

*Yaru Li and Mingming Chen contributed equally to this work.

Supplementary Figure S1

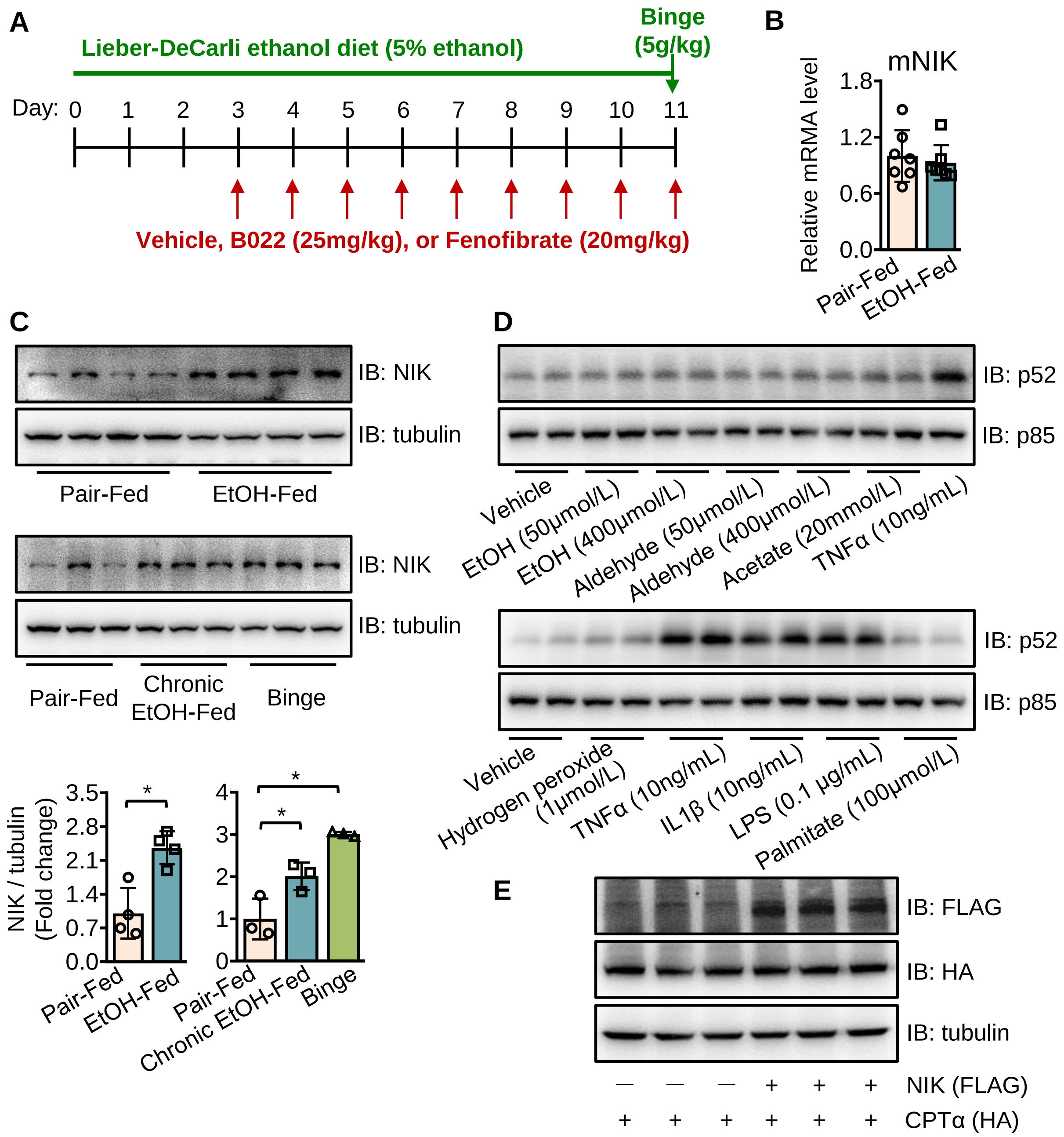

Figure S1. Chronic ethanol feeding and binge both upregulate hepatic NIK; Identification of the stimuli of NIK.

(A) Schematic representation of ALD mouse model replication. (B) Hepatic mRNA level of NIK in mice receiving a chronic-plus-binge ethanol diet (n = 7 for each group). (C) Representative immunoblots of NIK and tubulin in the livers of mice receiving chronic-plus-binge ethanol feeding, single chronic ethanol feeding or single binge. (D) Primary hepatocytes were treated with stimuli as indicated for 24 h after 16 h culture. Cell extracts were immunoblotted with antibodies against p52 or p85. (E) FLAG-tagged NIK and HA-tagged CPT1α were coexpressed in AML12 cells. Cell extracts were immunoblotted with antibodies against FLAG, HA, or tubulin. Values are demonstrated as means ± SEM. **P* <0.05, for comparisons with the control.

Supplementary Figure S2

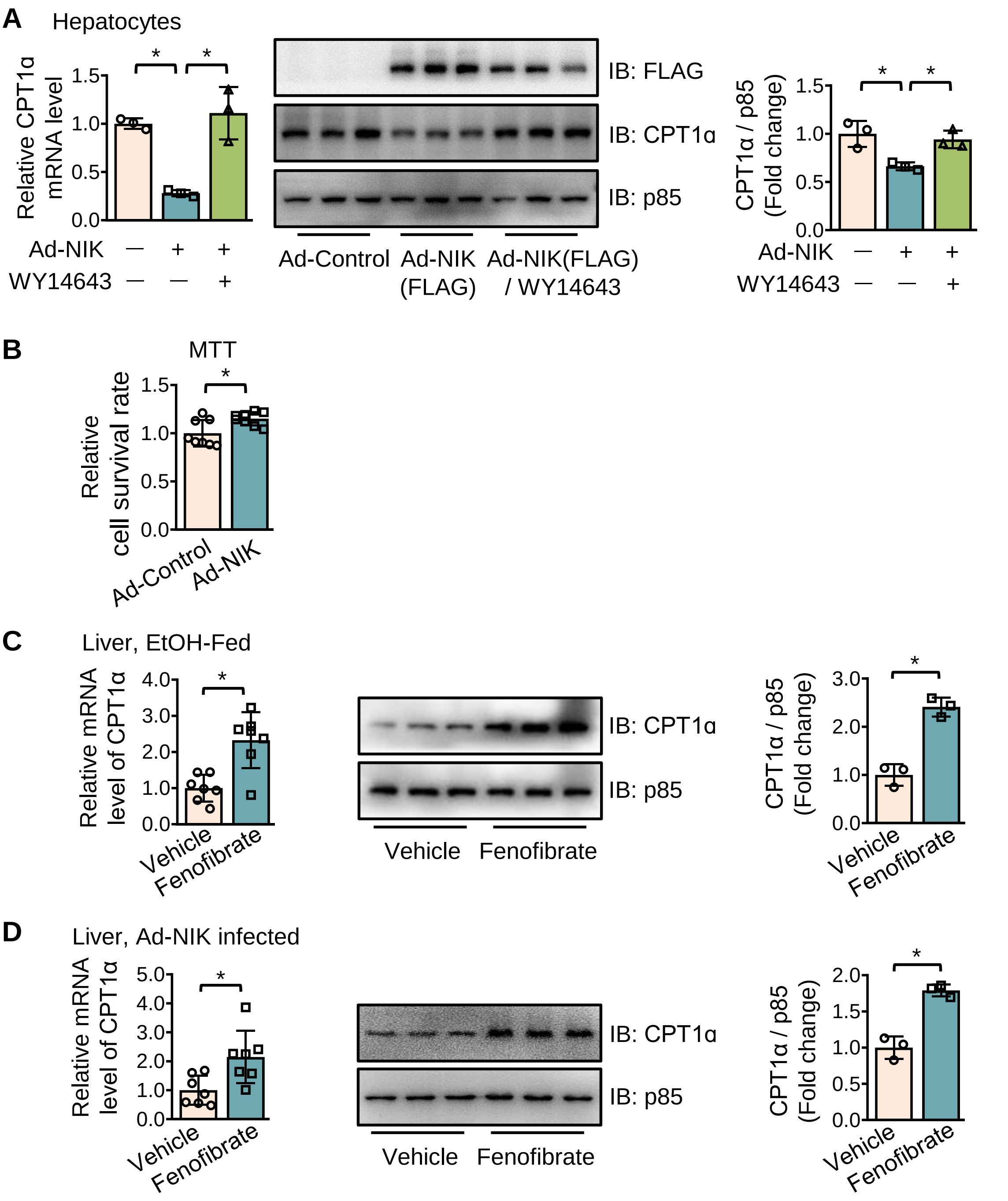

Figure S2. NIK-reduced expression of CPT1α is reversed by a PPARα agonist.
Hepatocytes were infected with NIK (Ad-NIK) or control (Ad-Control) adenoviruses, and exposed to WY14643 (5 μmol/L) or vehicle. (A) The mRNA and protein levels of CPT1α (n = 3 for each group). (B) MTT assay for cell viability (n = 8 for each group). (C) The mRNA and protein levels of CPT1α in the livers of WT mice fed with a chronic-plus-binge ethanol diet and treated with or without fenofibrate (20 mg/kg/day; n = 7 for each group). (D) The mRNA and protein levels of CPT1α in the livers of WT mice infected with adenoviruses expressing FLAG-tagged NIK and treated with or without fenofibrate (20 mg/kg/day, n = 7 for each group). Values are demonstrated as means ± SEM. *P <0.05, for comparisons with the control.

Supplementary Figure S3

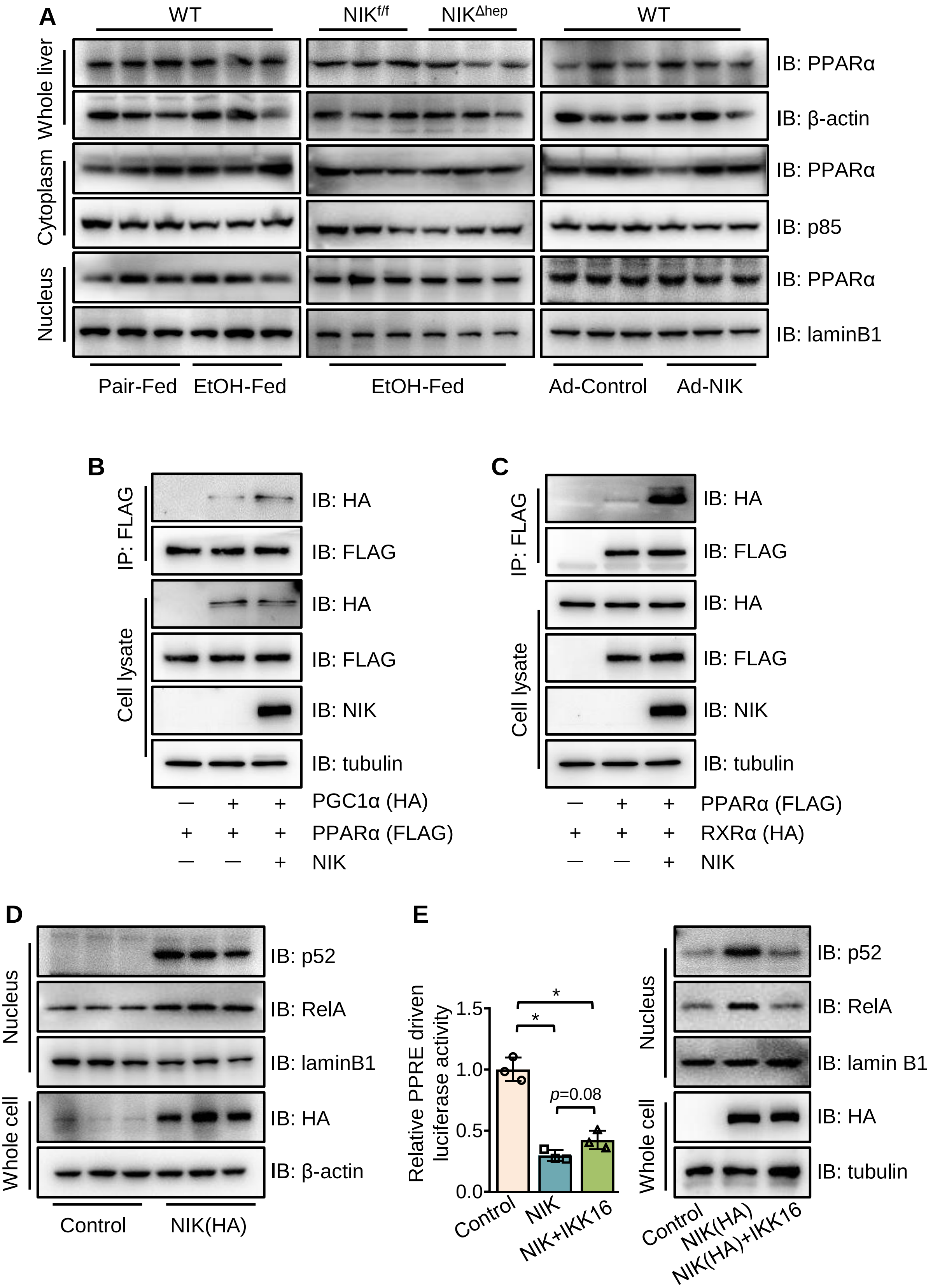

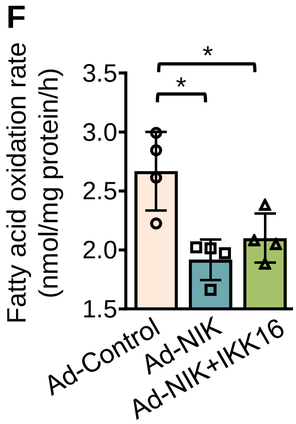

Figure S3. The effect of NIK on the protein level and nuclear translocation of PPARα and the interactions of PPARα with PGC1α and RXRα.

(A) The extracts of the whole liver, liver cytoplasm, and liver nucleus were immunoblotted with antibodies against PPARα, β-actin, p85 or lamin B1. WT mice were fed with a chronic-plus-binge ethanol (EtOH-fed) or control diet (Pair-fed; left panel). $NIK^{f/f}$ and $NIK^{\Delta hep}$ mice fed with a chronic-plus-binge ethanol diet (middle panel), WT mice infected by NIK or control adenoviruses for 5 d (right panel). (B) NIK, HA-tagged PGC1α, and FLAG-tagged PPARα were coexpressed in AML12 cells as indicated. Cell extracts were immunoprecipitated with anti-FLAG M2 affinity gel and immunoblotted with antibodies against HA, FLAG, NIK, or tubulin. (C) HA-tagged RXRα, NIK, and FLAG-tagged PPARα were coexpressed in AML12 cells as indicated. Cell extracts were immunoprecipitated with anti-FLAG M2 affinity gel and immunoblotted with antibodies against NIK, FLAG, HA, or tubulin. (D) AML12 cells transfected with a vector expressing HA-tagged NIK or a control vector. The extracts from cells or cellular nucleus were immunoblotted with antibodies against p52, RelA, lamin B1, HA, or β-actin. (E) Luciferase assays were performed to assess PPARα activity when PPARα and HA-tagged NIK is overexpressed, with or without the treatment of IKK16 (1 μmol/L; n = 3 for each group). The extracts from cells or cellular nucleus were immunoblotted with antibodies against p52, RelA, lamin B1, HA, or tubulin. (F) Hepatocytes infected with adenoviruses expressing FLAG-tagged NIK (Ad-NIK) with or without the treatment of IKK16 (1 μmol/L; n = 4 for each group). The fatty acid oxidation rates were determined. Values are demonstrated as means ± SEM. *P <0.05, for comparisons with the control.

Supplementary Figure S4

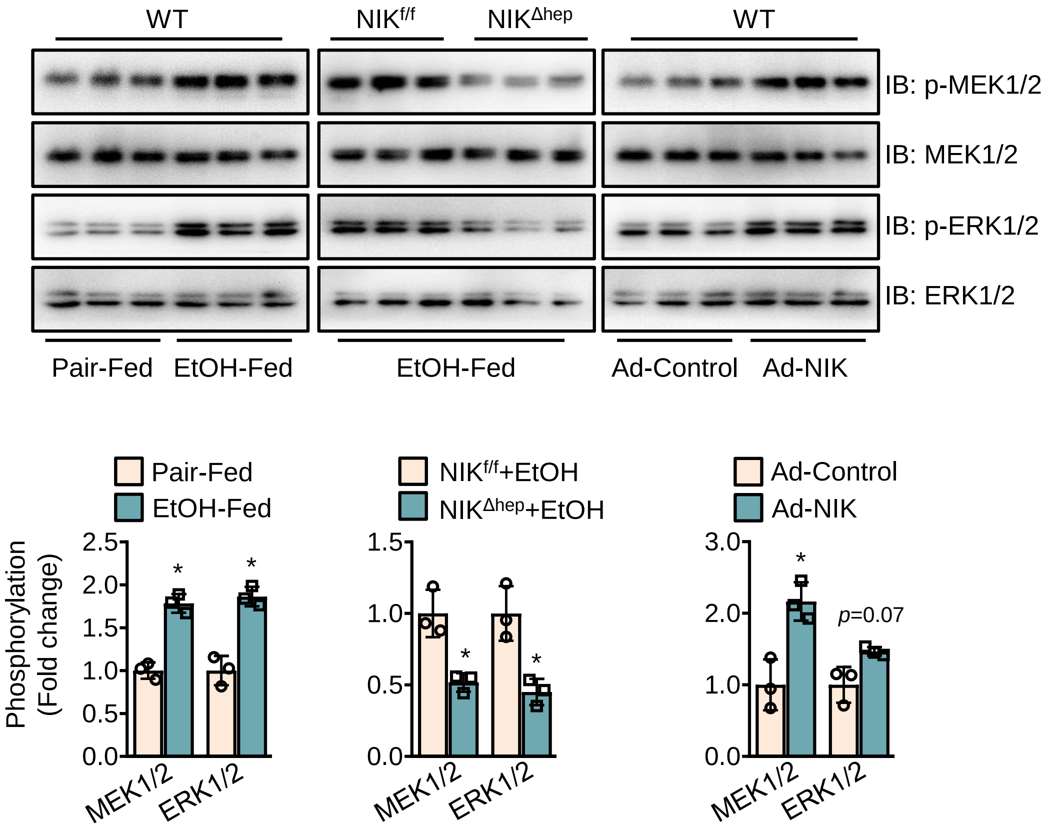

Figure S4. The effects of ethanol consumption and NIK activity on the phosphorylation levels of MEK1/2 and ERK1/2 in liver.

Liver extracts were immunoblotted with antibodies against p-MEK1/2, MEK1/2, p-ERK1/2, and ERK1/2. WT mice fed a chronic-plus-binge ethanol (EtOH-fed) or control diet (Pair-fed; left panel), $NIK^{f/f}$ and $NIK^{\Delta hep}$ mice fed a chronic-plus-binge ethanol diet (middle panel), and WT mice were infected with NIK (Ad-NIK) or control (Ad-Control) adenoviruses for 5 d (right panel). Values are demonstrated as means ± SEM. *P <0.05, for comparisons with the control.

Supplementary Figure S5

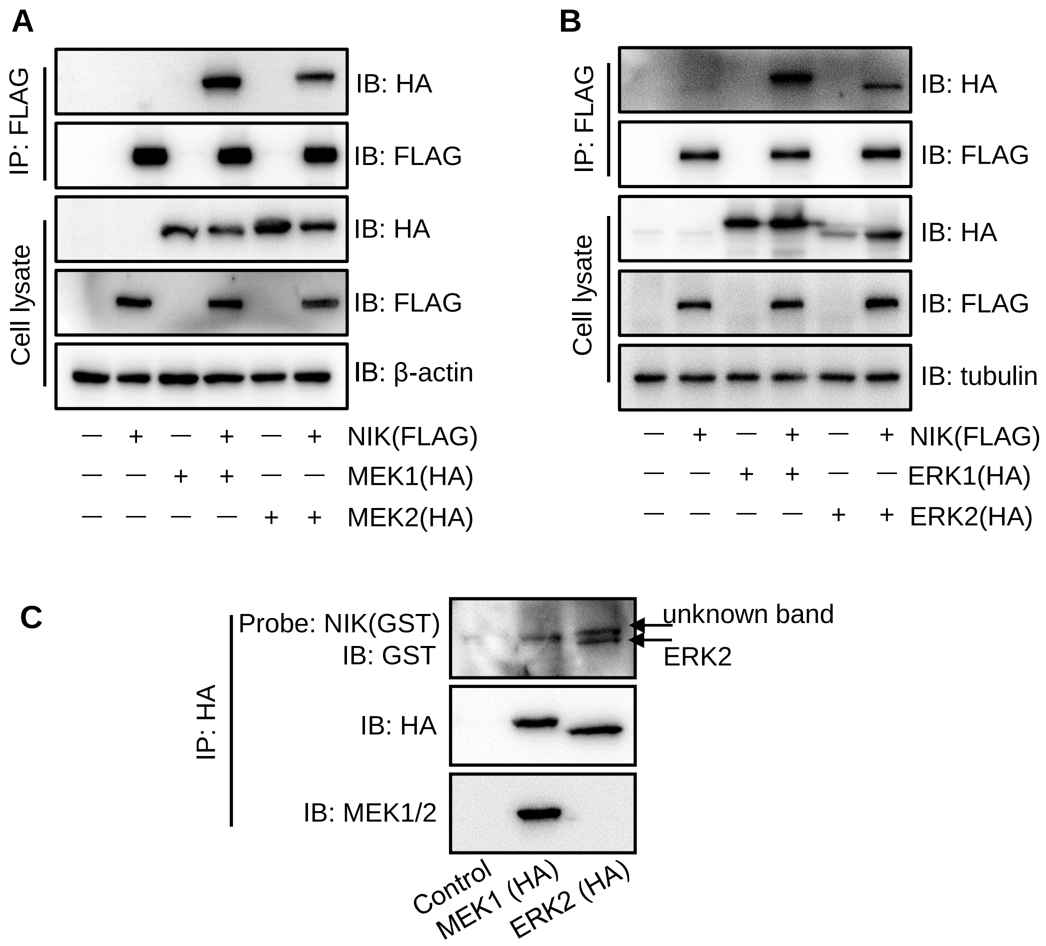

Figure S5. The interactions of NIK with MEK1/2 and ERK1/2.

(A) The extracts of AML12 cells expressing FLAG-tagged NIK and HA-tagged MEK1 or MEK2 were immunoprecipitated using anti-FLAG M2 affinity gel and immunoblotted with antibodies against HA, FLAG, and β-actin. (B) The extracts of AML12 cells expressing FLAG-tagged NIK and HA-tagged ERK1 or ERK2 were immunoprecipitated using anti-FLAG M2 affinity gel and immunoblotted with antibodies against HA, FLAG, and tubulin. (C) HA-tagged MEK1 and ERK2 expressed in AML12 cells were immunopurified using Pierce anti-HA agarose. The immunoprecipitates, separated by SDS-PAGE, were subjected to far-western blot analysis using GST-infused NIK as a probe and immunoblotted with antibodies against GST, HA, or MEK1/2.

Supplementary Figure S6

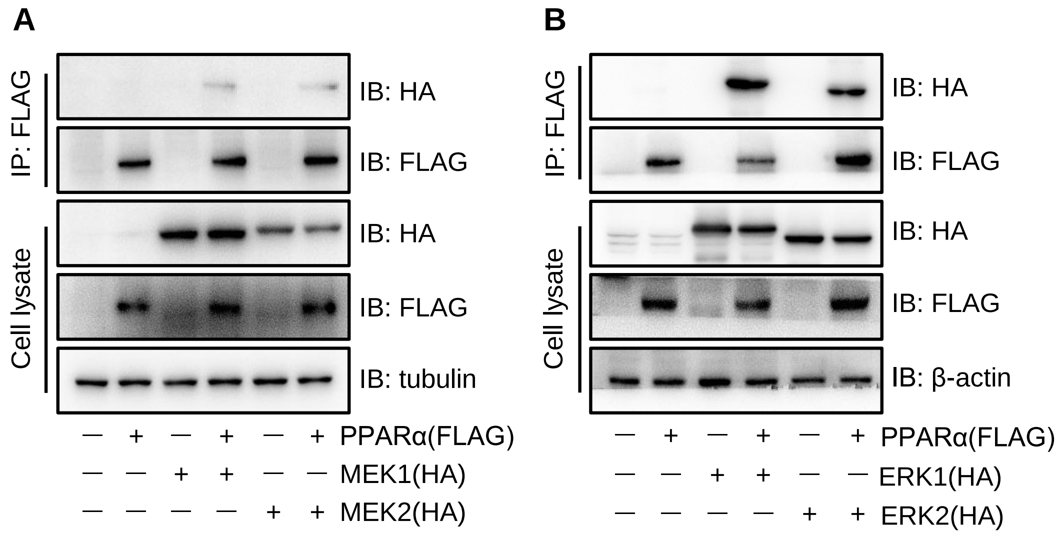

Figure S6. The interactions of PPARα with MEK1/2 and ERK1/2.
(A) Extracts of AML12 cells expressing FLAG-tagged PPARα and HA-tagged MEK1 or MEK2 were immunoprecipitated with anti-FLAG M2 affinity gel and immunoblotted with antibodies against HA, FLAG, and tubulin. (B) The extracts of AML12 cells expressing FLAG-tagged PPARα and HA-tagged ERK1 or ERK2 were immunoprecipitated with anti-FLAG M2 affinity gel and immunoblotted with antibodies against HA, FLAG, and β-actin.

Supplementary Figure S7

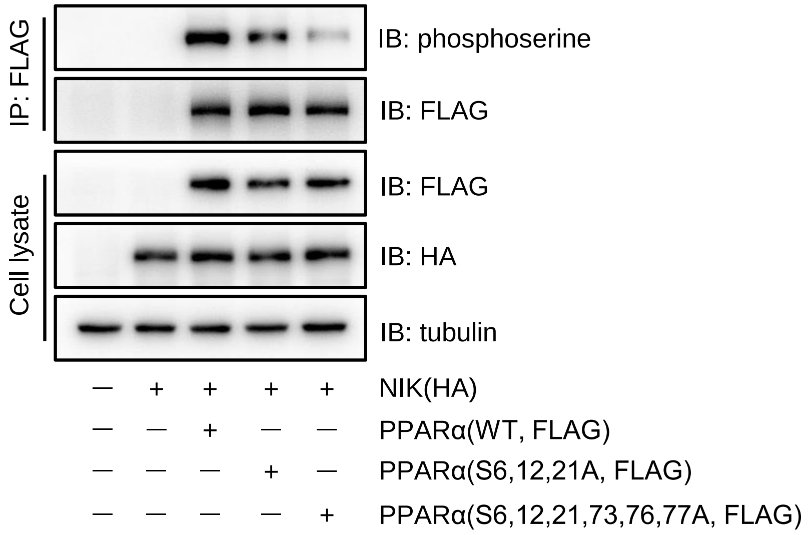

Figure S7. NIK-induced phosphorylation of PPARα.

Extracts of AML12 cells expressing HA-tagged NIK and FLAG-tagged PPARα and its mutants as indicated were immunoprecipitated with anti-FLAG M2 affinity gel and immunoblotted with antibodies against phosphoserine, HA, FLAG, and tubulin.

Supplementary Figure S8

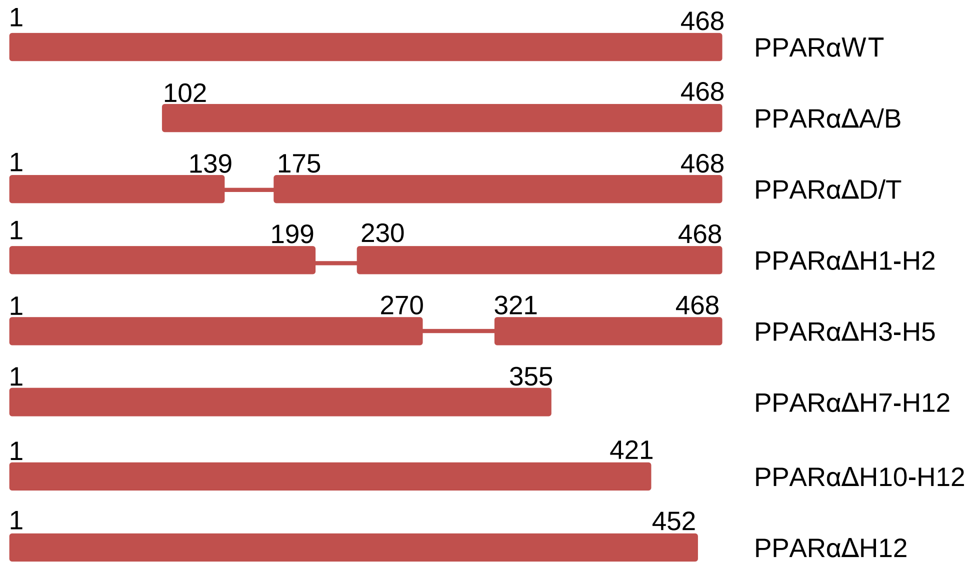

Figure S8. Schematic representation of different truncations of PPARα

Supplementary Figure S9

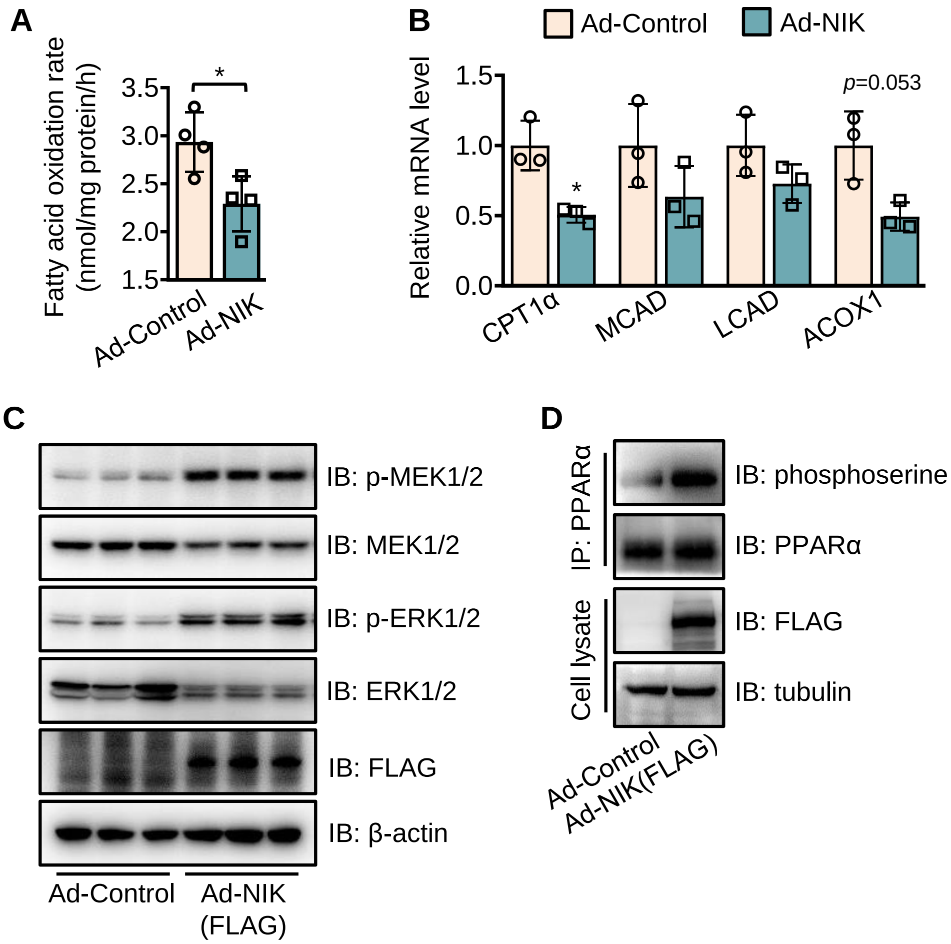

Figure S9. NIK suppresses fatty acid oxidation by MEK1/2-ERK1/2-PPARα pathway in HepG2 cell.

HepG2 cells were infected by adenoviral vectors expressing FLAG-tagged NIK (Ad-NIK) or control (Ad-Control) for 24 h. After serum starvation for 5 h, cells were subject to subsequent experiments. (A) Fatty acid oxidation rate was determined (n=4 for each group). (B) The mRNA levels of CPT1α, MCAD, LCAD, and ACOX1 were determined (n=3 for each group). (C) Representative immunoblotting of p-MEK1/2, MEK1/2, p-ERK1/2, ERK1/2, FLAG, and β-actin in cell lysates. (D) Cell extracts were immunoprecipitated with an anti-PPARα antibody and immunoblotted with antibodies against phosphoserine, PPARα, FLAG or tubulin. Values are demonstrated as means ± SEM. **P* <0.05, for comparisons with the control.

Table S1: The primers used for cloning.

Genes	Template	Target Vector	Primers (5'-3')	Sites
NIK,<br>NIK(KA)	pRK5-NIK<br>pRK5-NIK(KA)	pcDNA-HA3	GATCCCCCGGGCTGCAGGAATTC<br>ATGGCTGTGATGGAAATGGC	5'-EcoR1
			ATAGAATAGGGCCCCCCCTCGAG<br>TTAGGGTCGGTTCTCCAGCTGG	3'-Xho1
NIK,<br>NIK(KA)	pRK5-NIK<br>pRK5-NIK(KA)	pAdeno-TBG-<br>MCS-3Flag	GATCAGATCTCGAGCTCAAGCTT<br>ATGGCAGTGATGGAAATGGC	5'-Hind3
			TAGTACCGGTGAATTCGAAGCTT<br>CGGTCTGTTCTCCAGCTGGCC	3'-Hind3
NIK	pRK5-NIK	pcDNA3.1 (+)	ACTAGTCCAGTGTGGTGGAATTC<br>ATGGCTGTGATGGAAATGGC	5'-EcoR1
			TTAAACGGGCCCTCTAGACTCGA<br>GTTACGGACGGTTCTCCAGCTG	3'-Xho1
PPARα	pSG5 PPARα	p3XFlag -<br>CMV7.1	GATGACAAGCTTGCGGCCGCGAT<br>GGTGGACACAGAGAGC	5'-Not1
			GTACCAGATCTATCGATGTCAGT<br>ACATGTCTCTGT	3'-Not1
PPARα	pSG5 PPARα	pcDNA-HA3	GGATCCCCCGGGCTGCAGGAATT<br>CATGGTGGACACAGAGAGC	5'-EcoR1
			TATAGAATAGGGCCCCCCCTCGA<br>GTTAGTACATGTCTCTGT	3'-Xho1
PPARα<br>ΔA/B	pSG5 PPARα	pcDNA-HA3	GGATCCCCCGGGCTGCAGGAATT<br>CATGTGTCGAATATGTGGG	5'-EcoR1
			TATAGAATAGGGCCCCCCCTCGA<br>GTTAGTACATGTCTCTGT	3'-Xho1
PPARα<br>ΔD/T	pSG5 PPARα	pcDNA-HA3	GGATCCCCCGGGCTGCAGGAATT<br>CATGGTGGACACAGAGAGC	5'-EcoR1
			AGCTGGTGTACGACAAGTGTAGA<br>ATGCCAAGATCTGAAAA	
			TTTTCAGATCTTGGCATTCTACAC<br>TTGTCGTACACCAGCT	
			TATAGAATAGGGCCCCCCCTCGA<br>GTTAGTACATGTCTCTGT	3'-Xho1
PPARα<br>ΔH1-H2	pSG5 PPARα	pcDNA-HA3	GGATCCCCCGGGCTGCAGGAATT<br>CATGGTGGACACAGAGAGC	5'-EcoR1
			ACGACCTGAAAGATAGTGAAGCA<br>GGAAAGACCAGCAACAA	
			GTTGCTGGTCTTTCCTGCTTCACT<br>ATCTTTCAGGTCGT	
			TATAGAATAGGGCCCCCCCTCGA<br>GTTAGTACATGTCTCTGT	3'-Xho1
PPARα<br>ΔH3-H5	pSG5 PPARα	pcDNA-HA3	GGATCCCCCGGGCTGCAGGAATT<br>CATGGTGGACACAGAGAGC	5'-EcoR1
			AGGTCCTGTCCTCCTTGATGAAC<br>A	
			GACAGGACCTCTGCCTCTTTGTCT<br>T	
			TATAGAATAGGGCCCCCCCTCGA<br>GTTAGTACATGTCTCTGT	3'-Xho1
PPARα<br>ΔH7-H12	pSG5 PPARα	pcDNA-HA3	GGATCCCCCGGGCTGCAGGAATT<br>CATGGTGGACACAGAGAGC	5'-EcoR1
			TATAGAATAGGGCCCCCCCTCGA<br>GCTACATGATGTCACAGAACG	3'-Xho1

PPARα ΔH10-H12	pSG5 PPARα	pcDNA-HA3	GGATCCCCCGGGCTGCAGGAATTCATGGTGGACACAGAGAGC	5’-EcoR1
			TATAGAATAGGGCCCCCCCTCGAGTTATGGGAAGAGGAAGGTGT	3’-Xho1
PPARα ΔH12	pSG5 PPARα	pcDNA-HA3	GGATCCCCCGGGCTGCAGGAATTCATGGTGGACACAGAGAGC	5’-EcoR1
			TATAGAATAGGGCCCCCCCTCGAGCTAGGACTCGGTCTTCTTGA	3’-Xho1
PGC1α	pcDNA-f: PGC1	pcDNA-HA3	GATCCCCCGGGCTGCAGGAATTCTGTTCTCAAGACTCTGTATGG	5’-EcoR1
			ATAGAATAGGGCCCCCCCTCGAGTTACCTACGCAAGCTTCTCTG	3’-Xho1
ERK1	pMT ERK1	pcDNA-HA3	GTGGATCCCCCGGGCTGCAGGAATTCGCCGCCACCATGGCTCCG	5’-EcoR1
			ACTATAGAATAGGGCCCCCCCTCGAGGTGTCTGTTCTTGTTAGGG	3’-Xho1
ERK2	pCMV-myc-rERK2-MEK1_fusion	pcDNA-HA3	GTGGATCCCCCGGGCTGCAGGAATTCAACGAATTCAGATCTGGTACCA	5’-EcoR1
			ACTATAGAATAGGGCCCCCCCTCGAGTTAACTTCTGTATCCTGGCTG	3’- Xho1
ERK2	pCMV-myc-rERK2-MEK1_fusion	pCMV-3-tag-4A-myc	GGATCCCCCGGGTGCAGGAATTCGAATTCAGATCTGGTACCATGGCG	5’-EcoR1
			AGAGATGAGTTTCTGCTCCTCGAGACTTCTGTATCCTGGCTGG	3’-Xho1
ERK2	pCMV-myc-rERK2-MEK1_fusion	pAdeno-MCMV-MCS-3Flag	TCTCGAGCTCAAGCTTCGAATTCAGATCTGGTACCATGGCG	5’-EcoR1
			ATCGTCATCCTTGTAGTCGGATCCGCTTCTGTATCCTGGCTGG	3’-BamH1
MEK1	Mouse liver cDNA	pcDNA-HA3	GATCCCCCGGGCTGCAGGAATTCATGCCCAAGAAGAAGCCGACGC	5’-EcoR1
			GAATAGGGCCCCCCCTCGAGTCAGATGCTGGCAGCGTGGGTT	3’-Xho1
MEK2	Mouse liver cDNA	pcDNA-HA3	GATCCCCCGGGCTGCAGGAATTCATGCTGGCCCGGAGGAAGCCGG	5’-EcoR1
			GAATAGGGCCCCCCCTCGAGTCACACTGCAGTCCGCGTGGGT	3’-Xho1
MEK1	Mouse liver cDNA	pCMV-3-tag-4A-myc	GGAGCTCCACCGCGGTGGCGGCCGCATGCCCAAGAAGAAGCCGAC	5’-Not 1
			GAGGTCGACGGTATCGATAAGCTTTACGCCTGCTGCATGGGTTG	3’-Xho1

Table S2: The information concerning the antibodies and beads used.

Antibody	Company	Cat#	Species raised in	Mono/ polyclonal	Dilution
RelA	Cell signaling technology	8242	rabbit	monoclonal	1:1000
Phospho-ERK1/2	Cell signaling technology	4370	rabbit	monoclonal	1:1000
ERK1/2	Cell signaling technology	4695	rabbit	monoclonal	1:1000
Phospho-GSK3α/β	Cell signaling technology	9327	rabbit	monoclonal	1:1000
GSK3α/β	Cell signaling technology	5676	rabbit	monoclonal	1:1000
Phospho-JNK	Cell signaling technology	4668	rabbit	monoclonal	1:1000
JNK	Cell signaling technology	9252	rabbit	polyclonal	1:1000
Phospho-p38	Cell signaling technology	4511	rabbit	monoclonal	1:1000
p38	Cell signaling technology	9212	rabbit	polyclonal	1:1000
Phospho-MEK1/2	Cell signaling technology	9154	rabbit	monoclonal	1:1000
MEK1/2	Cell signaling technology	8727	rabbit	monoclonal	1:1000
RXRα	Cell signaling technology	3085	rabbit	monoclonal	1:1000
HA	Cell signaling technology	3724	rabbit	monoclonal	1:5000
Flag	Cell signaling technology	14793	rabbit	monoclonal	1:5000
Myc	Cell signaling technology	2276	mouse	monoclonal	1:5000
NIK	Cell signaling technology	4994	rabbit	polyclonal	1:1000
NF-κB (p52)	Santa Cruz Biotechnology	sc-7386	mouse	monoclonal	1:1000
CPT1α	Santa Cruz Biotechnology	sc-393070	mouse	monoclonal	1:1000
tubulin	Proteintech	10068-1-AP	rabbit	polyclonal	1:1000
β-actin	Proteintech	66009-1-Ig	mouse	monoclonal	1:1000
lamin B1	Abcam	ab16048	rabbit	polyclonal	1:1000
phosphoserine	Abcam	ab9332	rabbit	polyclonal	1:1000
PPARα	Abcam	ab24509	rabbit	polyclonal	1:1000
SUMO1	Abcam	ab32058	rabbit	monoclonal	1:1000
HRP-conjugated Affinipure Goat Anti-Mouse IgG(H+L)	Proteintech	SA00001-1	goat		1:5000

HRP-conjugated Affinipure Goat Anti-Rabbit IgG(H+L)	Proteintech	SA00001-2	goat		1:5000
Protein A/G-sepharose beads	7-Sea Biotech	P001-2			
Anti-FLAG M2 affinity gel	Sigma-Aldrich	A2220			
Pierce anti-HA agarose	Thermo Fisher Scientific	MA1-12455			

Table S3: The primers used for reverse transcriptional quantitative PCR.

**Genes**	**Species**	**Forward**	**Reverse**
CPT1α	mouse	CTGATGACGGCTATGGTGTTT	GTGAGGCCAAACAAGGTGATA
MCAD	mouse	ACCCTGTGGAGAAGCTGATG	AGCAACAGTGCTTGGAGCTT
LCAD	mouse	CACTCAGATATTGTCATGCCCT	TCCATTGAGAATCCAATCACTC
ACOX1	mouse	TAACTTCCTCACTCGAAGCCA	AGTTCCATGACCCATCTCTGTC
NIK	mouse	TGTGGGAAGTGGGAGATCCTA	GGCTGAACTCTTGGCTATTCTCA
RPLP0	mouse	AAGCGCGTCCTGGCATTGTCT	CCGCAGGGGCAGCAGTGGT
CPT1α	human	ATCAATCGGACTCTGGAAACGG	TCAGGGAGTAGCGCATGGT
MCAD	human	TGGATAACCAACGGAGGAAAAG	CTGGGGTATCTGCTTCCACA
LCAD	human	TGCAATAGCAATGACAGAGCC	CGCAACTACAATCACAACATCAC
ACOX1	human	AATCGGGACCCATAAGCCTTT	GGGAATACGATGGTTGTCCATTT
GADPH	human	CTGGGCTACACTGAGCACC	AAGTGGTCGTTGAGGGCAATG
